# Immune activation of primary human macrophages is suppressed by the coordinated action of *Yersinia* effectors

**DOI:** 10.1128/mbio.02547-25

**Published:** 2025-11-24

**Authors:** Indra Bekere, Sören Rob, Jonas Lübbe, Susanne Kulnik, Laura Berneking, Jiabin Huang, Marie Schnapp, Björn-Philipp Diercks, Andreas H. Guse, Alexander Carsten, Klaus Ruckdeschel, Martin Aepfelbacher

**Affiliations:** 1 Institute of Medical Microbiology, Virology and Hygiene, University Medical Center Hamburg-Eppendorf (UKE) 538358, Hamburg, Germany; 2 The Calcium Signalling Group, Department of Biochemistry and Molecular Cell Biology, University Medical Center Hamburg-Eppendorf 235709, Hamburg, Germany; Washington University in St. Louis, St. Louis, Missouri, USA

**Keywords:** *Yersinia*, macrophages, Yop, inflammation, inflammasome, epigenetics, RNA-seq, effectors, calcium signalling, inflammatory gene expression, bacterial effector networks

## Abstract

To suppress the host immune response, numerous bacterial pathogens utilize a type 3 secretion system (T3SS) that injects effector proteins into host target cells. We investigated the T3SS effectors of *Yersinia enterocolitica* (Yops) for their individual and combined effects on gene expression, inflammasome formation, and calcium signaling in primary human macrophages. YopP efficiently suppressed the upregulation and downregulation of thousands of macrophage genes induced by the bacteria’s inflammatory stimuli. This was accompanied by parallel changes in histone 3-serine 10 phosphorylation, which could represent an overarching regulatory mechanism of rapid gene expression in macrophage inflammatory response. Surprisingly, YopM and YopQ counteracted selected YopP effects on gene expression, for example, of cytokine pathways. A combination of YopP and YopQ, but not other combinations of Yops or any single Yop, reduced inflammasome formation. YopH alone blocked calcium transients in infected macrophages. We propose that the T3SS effectors of *Yersinia* antagonistically, synergistically, or individually subdue major immune pathways of human macrophages to jointly suppress macrophage activation.

**IMPORTANCE:**

Macrophages are one of the key target cells of pathogenic *Yersinia*, where central immune response pathways, such as phagocytosis, gene expression, and inflammasome assembly, are suppressed by secreted bacterial effectors (Yops) in a highly coordinated fashion. Most studies analyzing cooperation between Yop proteins have utilized cell lines and mouse-derived macrophages, which strongly differ from human macrophages. This study employed primary human macrophages and analyzed cooperation between different *Yersinia enterocolitica* effector proteins on gene expression, histone phosphorylation, calcium signaling, and inflammasome assembly. We reveal synergistic, antagonistic, and individual roles of different Yersinia effector proteins. This work highlights how highly coordinated activities of a limited set of effectors can efficiently disarm macrophage immune responses and lead to a successful infection.

## INTRODUCTION

Bacterial pathogens have evolved numerous strategies that efficiently suppress the antibacterial activities of the host’s immune system to support their pathogenicity. Gram-negative bacteria often inactivate immune cells using specialized machineries called secretion systems, of which Type III, IV, and VI secretion systems (T3SS/T4SS/T6SS) are the most widely distributed (1–3). These secretion systems inject effector proteins, which alone or in combination manipulate immune cell functions including cytoskeletal dynamics, for example, to prevent phagocytosis or cell-intrinsic surveillance mechanisms, for example, to suppress expression of inflammatory genes or block induction of cell death (4–6).

Pathogenic species of the Gram-negative bacteria, *Yersinia*, *Escherichia coli*, *Salmonella,* and *Shigella,* mainly use T3SSs to inject effector proteins into human target cells (2, 7). These effectors manipulate central cellular pathways through different molecular mechanisms (2, 8). Several studies have shown that the effectors act individually but also cooperatively on host processes (9–11).

Enteropathogenic *Y. enterocolitica* and *Y. pseudotuberculosis,* as well as the plague agent *Y. pestis,* suppress immune cell functions through seven T3SS effectors named YopE, YopT, YopH, YopO/YpkA, YopP/YopJ, YopM, and YopQ/YopK (second names are *Y. pseudotuberculosis*/*Y. pestis* homologs) (12). YopE, YopT, and YopO/YpkA inhibit phagocytosis and related cytoskeleton-dependent cell functions like chemotaxis by interfering with the activities of Rho GTP-binding proteins (13). YopH is a highly active tyrosine phosphatase that dephosphorylates signaling and cytoskeleton-associated proteins, thereby inhibiting phagocytosis in macrophages and Ca^2+^ signaling in lymphocytes and neutrophils (14–16). YopP/J, YopM, and YopQ/K downregulate major inflammatory pathways in innate immune cells (17–19). YopP/J acetylates and inhibits components of NF-κB and MAPK pathways, thus suppressing production of pro-inflammatory mediators and triggering cell death in macrophages (18, 20). YopM associates with RSK serine/threonine kinases (p90 ribosomal S6 kinase 1; MAPKAP-K1) and PKN kinases (protein kinase N; protein kinase C-related kinase/PRK) in the host cell nucleus and triggers activation of the JAK-STAT pathway to induce the production of interleukin-10 (IL-10) (21–23). YopQ/K has been shown to regulate the injection of T3SS effectors from within host cells by an ill-defined mechanism (24).

Here, we evaluated the individual and cooperative activities of the *Y. enterocolitica* effectors on major antibacterial immune pathways in primary human macrophages: (i) inflammatory gene expression; (ii) inflammasome activation; and (iii) increases in intracellular Ca^2+^ concentration. Primary human macrophages mirror the functions of the corresponding immune cells in the human body well. *Y. enterocolitica*-mediated initiation and execution of human macrophage cell death are considerably retarded compared to murine macrophages. Accordingly, first signs of cell death, indicated by caspase-8 activation, appear in human macrophages after around 3–5 h (25). This excludes a major influence of cytotoxic processes on the macrophage immune functions investigated here (26–28).

In earlier studies using gene microarrays, YopP was shown to efficiently suppress the induction of hundreds of genes in mouse macrophages (29, 30). In a more recent study using RNA sequencing technologies, YopP was found to affect thousands of epigenetic histone modifications at inflammatory genes in the human macrophage genome (28). In the past microarray study, YopM was reported to only minimally or not at all affect gene expression (29, 30). However, recent work demonstrated that YopM upregulates the expression of multiple genes of the JAK-STAT pathway, including IL-10, in human macrophages (22, 23). Until now, the effect of YopQ/YopK on gene expression has not been reported.

YopP/J, YopM, and YopQ/K have all been shown to suppress inflammasome activation in macrophages by different mechanisms. Canonical inflammasomes are cytoplasmic assemblies in which NOD-like receptors (NLRs), acting as sensors of pathogen-associated molecular patterns (PAMPs), activate the enzyme caspase-1 via the adapter protein apoptosis-associated speck-like protein containing a CARD (ASC). Caspase-1 cleaves preforms of the pore-forming molecule gasdermin D (GSDMD) and the cytokines IL-1β and IL-18, which leads to the cellular release of the cytokines through the GSDMD membrane pores formed and an inflammatory cell death known as pyroptosis (31). YopP/YopJ inhibits the expression of the caspase-1 substrates pro-IL1β and pro-IL18 through downregulation of NF-κB and MAPK signaling, although it may also mediate inflammasome-independent activation of caspase-1 (32). YopM blocks the pyrin inflammasome, which is first activated by the suppression of Rho-GTP-binding protein activity, for example, caused by YopE, YopT, or YopO/YpkA (33–35). This provides a vivid example of effector-triggered immunity that is counteracted by another effector (11). YopQ inhibits NLRP3 and NLRC4 inflammasomes, whose activation is triggered by multiple signaling molecules or constituents of the *Yersinia* T3SS, respectively (33, 36, 37). In lipopolysaccharide (LPS)-primed mouse macrophages, YopM and YopP/YopJ were found to cooperatively inhibit caspase-1 activation and IL-1β secretion (32, 38). Almost all studies on the *Yersinia* effects on inflammasome activity were done in mouse macrophages or mouse infection models (17, 18). However, it is known that triggers, regulatory mechanisms, and accessory proteins of canonical inflammasomes differ between mouse and human macrophages. For instance, activation of NLRP3 inflammasomes requires pretreatment with LPS in mouse but not in human macrophages and has a different structural basis in these species (31, 39). Interestingly, a recent study demonstrated that a *Y. pseudotuberculosis* mutant lacking all T3SS effectors activates the caspase-4 non-canonical inflammasome in human macrophages and intestinal cells and that this caspase-4 activation could be inhibited by cooperation of the T3SS effectors YopE, YopH, and YopK (*Y. pseudotuberculosis* homolog of YopQ) (40).

Intracellular Ca²^+^ signaling is involved in a variety of macrophage functions, including the control of gene transcription and the activation of the inflammasome (41). Even though macrophages are the most important target cells of pathogenic yersiniae, the effect of Yops on Ca^2+^ signaling in these cells has not been reported.

We report here that in primary human macrophages, *Yersinia* effectors act antagonistically in regulating inflammatory gene expression (YopP vs YopM/YopQ), cooperatively in inhibiting inflammasomes (YopP and YopQ), and individually in suppressing intracellular Ca^2+^ transients (YopH).

## RESULTS

### Global and time-dependent transcriptional changes in primary human macrophages infected with *Y. enterocolitica*

**Fig 1:**
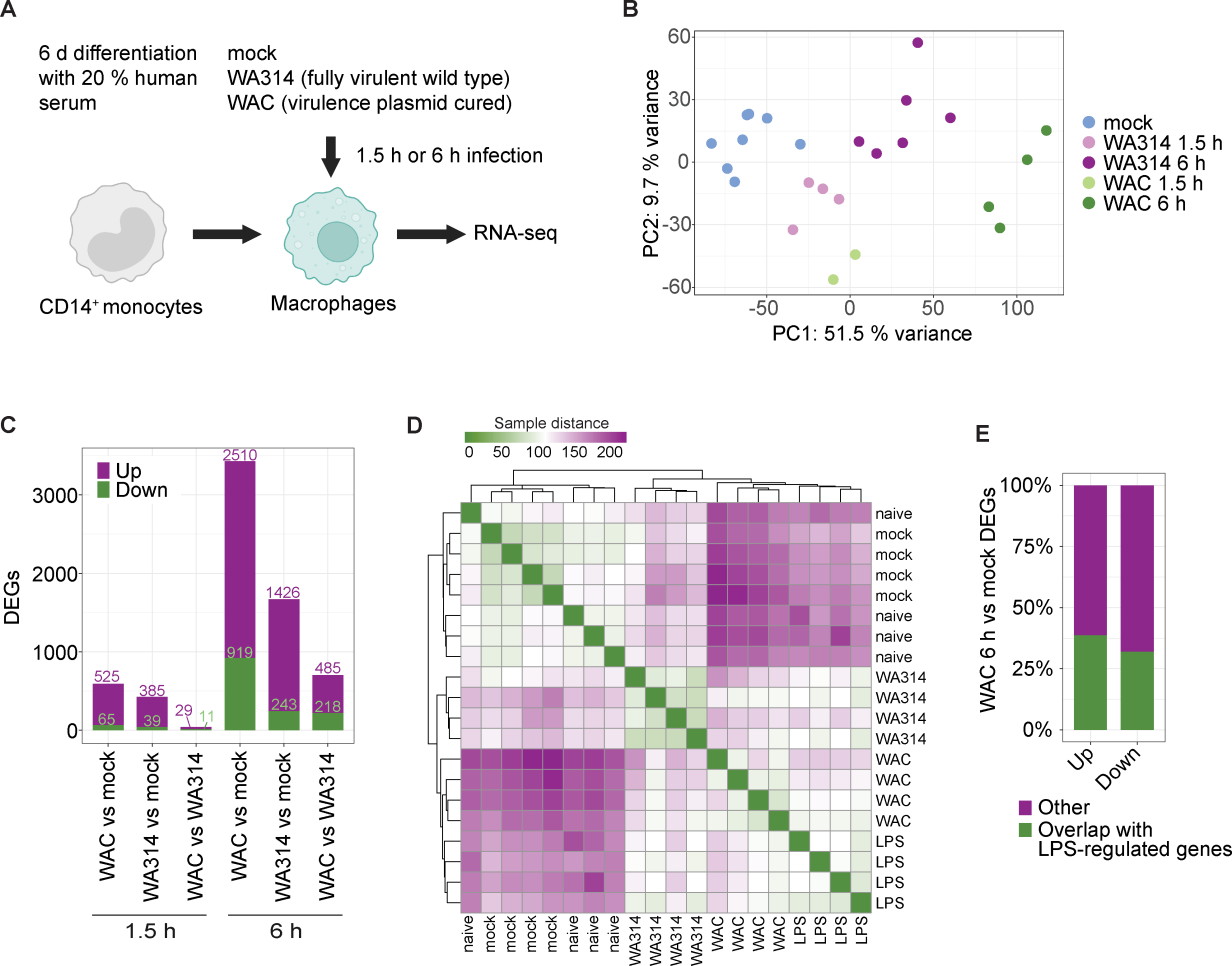
Global RNA expression analysis of *Y. enterocolitica*-infected primary human macrophages. (**A**) Experimental design. Human CD14^+^ monocytes were differentiated into macrophages by cultivation with 20% human serum for 6 ± 1 days. Macrophages from independent donors were mock infected or infected with avirulent *Y. enterocolitica* strain WAC or wild-type strain WA314 with a multiplicity of infection (MOI) of 100 for 1.5 h or 6 h and subjected to RNA-seq analysis. All samples analyzed in this study are listed in Table S2. (**B**) Principal component (PC) analysis of RNA-seq vst gene counts of all differentially expressed genes (DEGs) identified in this study (Tables S3 and S4). (**C**) Number of upregulated and downregulated DEGs for indicated comparisons and infection times (Table S3). (**D**) Heatmap representation of sample distance in human macrophages mock-infected or infected with indicated strains (6 h) combined with respective publicly available data from naive and LPS-stimulated macrophages (42, 43). (**E**) Percentage of DEGs from WAC vs mock-infected human macrophages that overlap with LPS-regulated genes obtained from published data (42, 43).

First, we investigated global changes in RNA expression in primary human macrophages infected with a virulent (WA314) and a virulence plasmid cured/avirulent (WAC) *Y. enterocolitica* strains after early 1.5 h and late 6 h time points of infection (Fig. 1A). RNA expression analysis (RNA-seq) was performed using next-generation sequencing (Materials and Methods) integrating previous and new data sets (23, 28). Because the avirulent strain WAC lacks the T3SS, it was used to determine the effects of the PAMPs and other bacterial inflammatory stimulators. Usage of the wild-type strain WA314 allowed us to determine the effect of the T3SS effectors on the inflammatory stimulator-induced gene expression changes.

Principal component analysis (PCA) of the RNA counts showed clustering of the biological replicates, which indicates high reproducibility of the experiments (Fig. 1B). After 1.5 h of infection, the clusters of mock, WAC, and WA314 infection were located closer to each other than after 6 h of infection, indicating a stronger and more pronounced transcriptional response at 6 h. Clearly separated clusters for WAC and WA314 after 6 h indicate a significant modulation of the bacterial mediator-induced gene expression by the T3SS effectors (Fig. 1B).

After 1.5 h of infection, only slightly higher numbers of differentially expressed genes (DEGs, log2 fold change ≥ [2] and *P*-adjusted ≤0.01) were found in the WAC vs mock group as compared to the WA314 vs mock group (Fig. 1C; Table S3). At this infection time, only a few genes were upregulated and downregulated in the WAC vs WA314 group (Fig. 1C). This suggests that although already after 1.5 h of infection, a significant transcriptional response is caused by the *Yersinia* inflammatory mediators, only a limited T3SS effector activity is detectable at this time point.

After 6 h of infection, almost six times more DEGs than after 1.5 h were altogether identified (Fig. 1C; Table S3). WAC vs mock showed a considerably larger number of DEGs than WA314 vs mock. This suggests that the inflammatory response of the macrophages to *Yersinia* infection is strongly repressed by the T3SS effectors after 6 h (Fig. 1C). As seen in the WAC vs WA314 group, the *Yersinia* T3SS effectors altogether modulated the expression of 703 genes (upregulation and downregulation of 485 and 218 genes, respectively; Fig. 1C).

We evaluated to what extent gene expression profiles from human macrophages not infected (mock) or infected with WAC or WA314 for 6 h overlapped with naïve and LPS-treated macrophages (42, 43). For this, we employed publicly available RNA-seq data sets from naïve and *E. coli* LPS-stimulated primary human macrophages (42, 43) and compared them with our data set in a sample-to-sample distance heatmap (Fig. 1D). Three groups emerged: (i) naive macrophages and mock; (ii) WA314; and (iii) WAC and LPS (Fig. 1D). That the WA314 group clearly separated from the WAC/LPS group reflects the extensive modulation of the bacterial component/LPS-induced effects by the T3SS effectors (18). On a quantitative level, 38.8% and 32.1% of the genes upregulated and downregulated by WAC in macrophages of our study were also upregulated and downregulated, respectively, by LPS in human macrophages (Fig. 1E). These data indicate that the *Yersinia*-induced transcriptional changes are to a large degree caused by LPS (44). The remaining changes must then be caused by other bacterial stimulators and components, such as PAMPs, surface adhesins, and constituents of the T3SS or by T3SS effector activities (36, 44, 45).

### Transcriptional profiles and enriched pathways in human macrophages after 1.5 h and 6 h of *Yersinia* infection

**Fig 2:**
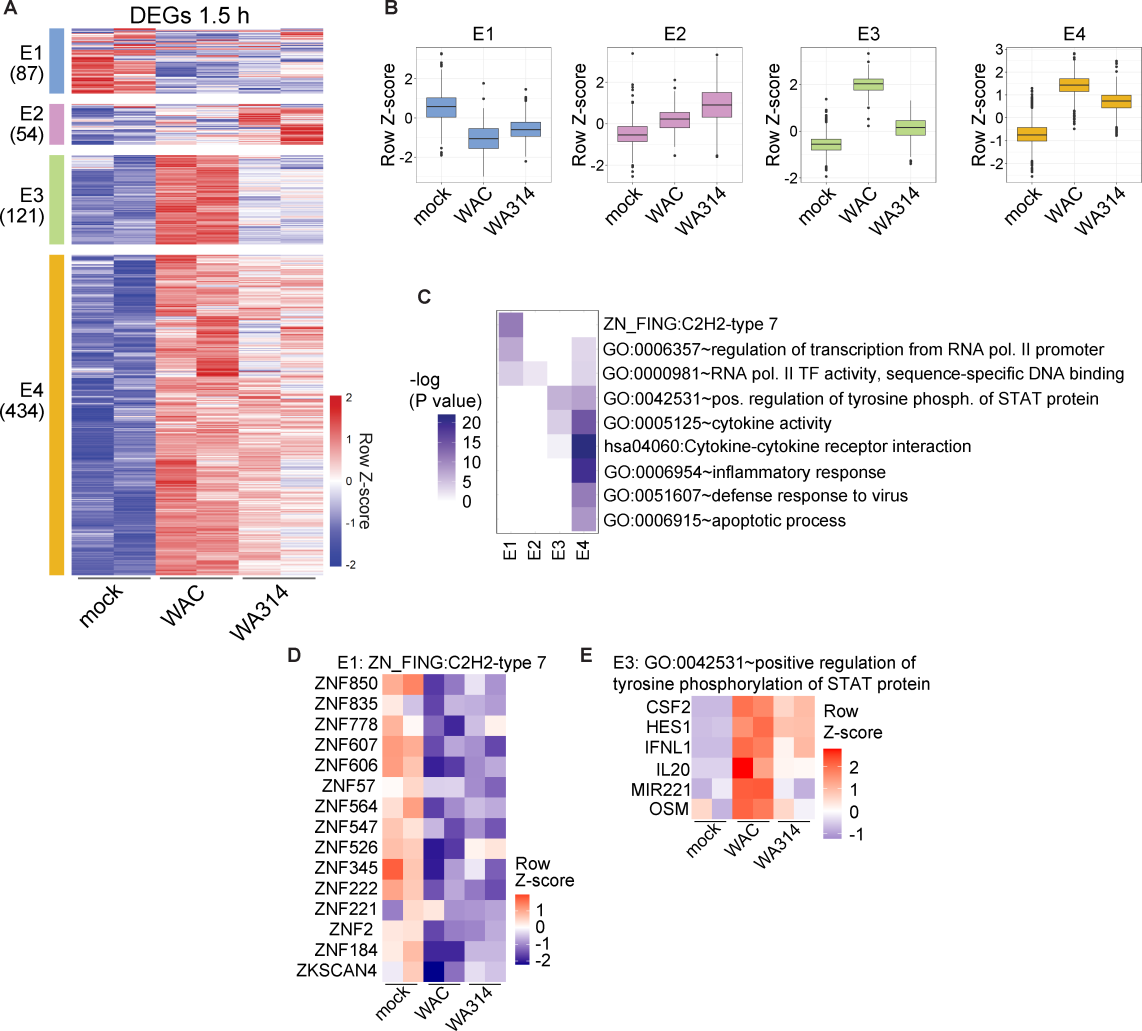
RNA expression profiles and enriched pathways in human macrophages infected for 1.5 h with *Y. enterocolitica*. (**A**) Clustered heatmap of DEGs (defined as log2 fold change equal to or larger than 2 and adjusted *P*-value equal to or smaller than 0.01) in comparisons between mock-, WAC-, and WA314 infected macrophages after 1.5 h of infection. Gene vst counts were row-scaled (row Z-score). Four major clusters, E1–E4, were identified (number of genes in brackets). Two representative biological replicates are shown for each condition with all replicates shown in Fig. S1A. (**B**) Boxplots of row-scaled vst counts for genes from clusters E1–E4 (**A**) showing the global expression profile in each cluster when taking into account all replicates. (**C**) Heatmap showing –log10 transformed *P*-values and enriched pathways for clusters E1–E4 (**A**). (**D**) Heatmap of row-scaled vst counts for genes enriched in cluster E1 that belong to C2H2 type Zinc finger type 7. Two representative biological replicates are shown, with all replicates depicted in Fig. S1B. (**E**) Heatmap of row-scaled vst counts for genes enriched in cluster E3 that belong to the pathway, positive regulation of tyrosine phosphorylation of STAT protein. Two representative biological replicates are shown, with all replicates depicted in Fig. S1D.

After 1.5 h of infection, 696 unique DEGs were found between mock-, WAC-, and WA314 infected macrophages that could be assigned to the four clusters Early-1 to Early-4 (E1-E4; Fig. 2A and B; Fig. S1A; Tables S4 and S5). E1 genes were downregulated by both WAC and WA314 as compared to mock and were enriched in regulation of transcription with 17% of the transcripts belonging to the Zinc finger C2H2-like group (Fig. 2B through D; Fig. S1B) (46). E2 transcripts were upregulated by WA314 and WAC when compared to mock and were also enriched in transcriptional activity and included numerous central transcription factors (Fig. 2B and C; Fig. S1C). The transcripts in clusters E3 and E4 were strongly upregulated by WAC, and this was inhibited by WA314, more so in E3 than in E4 (Fig. 2A and B). E3 transcripts contain genes involved in the regulation of STAT protein phosphorylation (Fig. 2C and E; Fig. S1D). Transcripts in the large cluster E4 were associated with inflammatory response pathways such as cytokine-cytokine receptor interaction and apoptotic process (Fig. 2C; Fig. S1E and F) and were consequently enriched in motifs for transcription factors RHD/NF-κB, TBP, and IRF (Fig. S1G). NF-κB is the main mediator of inflammatory gene expression, whereas IRFs regulate the expression of secondary response genes. IRFs themselves are induced in an autocrine/paracrine fashion by type I IFNs (47, 48). We conclude that an early effect of the inflammatory mediators of *Y. enterocolitica* is to downregulate or upregulate transcriptional regulators. Furthermore, E3 and E4 transcripts are primary and secondary inflammatory response genes that are induced already after 1.5 h of infection by the bacterial mediators and are already significantly (E3) or only slightly (E4) downregulated by the T3SS effectors.

**Fig 3:**
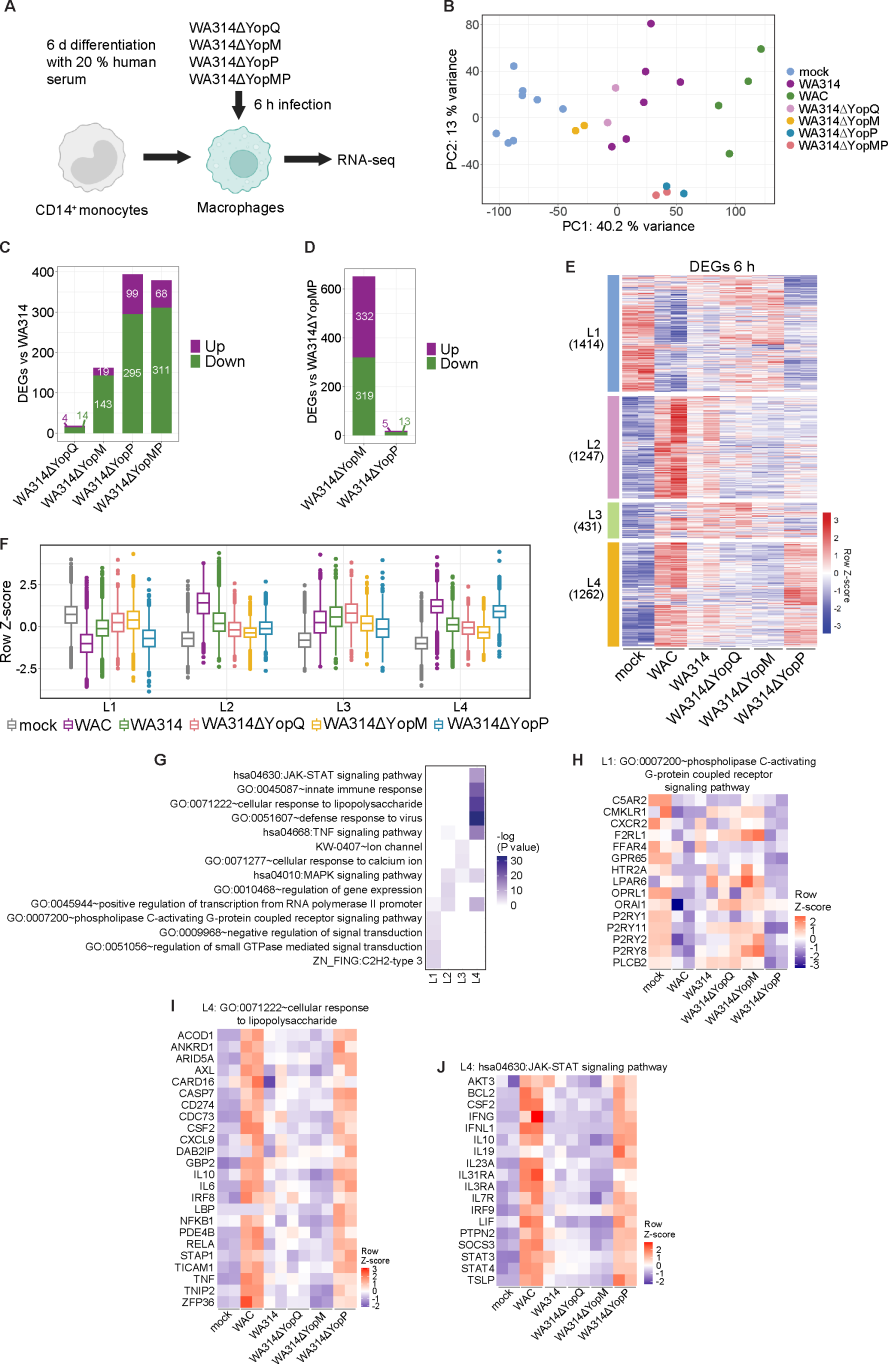
RNA expression profiles and enriched pathways in human macrophages infected for 6 h with *Y. enterocolitica*. (**A**) Experimental setup. CD14^+^ monocytes were differentiated into macrophages by cultivation with 20% human serum for 6 ± 1 days. Macrophages from ≥ two independent donors were infected with the Yop-mutant strains WA314ΔYopM, WA314ΔYopP, WA314ΔYopMP, or WA314ΔYopQ with an MOI of 100 for 6 h and subjected to RNA-seq analysis. (**B**) PCA of vst gene counts of all DEGs identified in this study (Tables S3 and S4). (**C**) Number of upregulated and downregulated DEGs in comparisons of WA314ΔYopQ, WA314ΔYopM, WA314ΔYopP, and WA314ΔYopMP vs WA314 (Table S3). (**D**) Number of upregulated and downregulated DEGs in comparisons between WA314ΔYopM and WA314ΔYopP vs WA314ΔYopMP (Table S3). (**E**) Clustered heatmap of DEGs for comparisons between mock-, WAC-, WA314-, WA314ΔYopQ-, WA314ΔYopM-, and WA314ΔYopP-infected macrophages after 6 h. Gene vst counts were row-scaled (row Z-score). Clusters L1–L4 were identified (number of genes in brackets). Two representative replicates are shown with all replicates and WA314ΔYopMP shown in Fig. S2A. (**F**) Boxplots of row-scaled vst counts for genes from clusters L1–L4 (**E**) showing the global expression profile in each cluster when taking into account all replicates. (**G**) Heatmap showing −log10 transformed *P*-values and enriched pathways for clusters L1–L4. (**H**) Heatmap of row-scaled vst counts for genes enriched in cluster L1 that belong to phospholipase C-activating G-protein-coupled receptor signaling pathway. Two representative biological replicates are shown, with all replicates depicted in Fig. S2B. (**I**) Heatmap of row-scaled vst counts for selected genes enriched in cluster L4 that belong to the cellular response to LPS pathway. Two representative biological replicates are shown, with all replicates and genes depicted in Fig. S2I. (**J**) Heatmap of row-scaled vst counts for selected genes enriched in cluster L4 that belong to the JAK-STAT signaling pathway. Two representative biological replicates are shown, with all replicates and genes depicted in Fig. S2J.

To define the individual roles and potential interplay of the immunomodulatory T3SS effectors YopP, YopM, and YopQ on regulation of gene expression in macrophages, we analyzed gene expression changes from macrophages infected for 6 h with strains deficient in the respective Yops WA314ΔYopQ, WA314ΔYopM, WA314ΔYopP, and WA314ΔYopMP using previous and newly generated data sets (23, 28) (Table S2; Fig. 3A). PCA of the RNA-seq data showed that the WA314ΔYopP and WA314ΔYopMP clusters located clearly separate, whereas the WA314ΔYopQ and WA314ΔYopM clusters located close to the WA314 cluster (Fig. 3B). This indicates a stronger effect of YopP than of YopQ and YopM on gene transcription. Consistent with this, roughly twice as many DEGs were detected in the WA314ΔYopP vs WA314 than in the WA314ΔYopM vs WA314 comparison, and only a small number of DEGs were seen in the WA314ΔYopQ vs WA314 comparison (Fig. 3C; Table S3). Furthermore, the number of DEGs for WA314ΔYopP vs WA314 and WA314ΔYopMP vs WA314 was about the same (Fig. 3C), and the number of DEGs for WA314ΔYopP vs WA314ΔYopMP was negligible (Fig. 3D). We conclude that YopP has the greatest, YopM a medium, and YopQ a minor effect on inflammatory mediator-induced gene transcription. Moreover, a comparison of WA314ΔYopM vs WA314ΔYopMP indicates that there is no additional effect when YopM is deleted in the absence of YopP (Fig. 3D). Therefore, the effects of the WA314ΔYopMP strain were not further included in the main results (see supplemental figures for these results). The altogether 4,354 unique DEGs in macrophages infected with the different *Yersinia* strains could be assigned to four clusters termed Late-1 to Late-4 (L1–L4) (Fig. 3E; Fig. S2A; Table S6). L1 transcripts were downregulated by the bacterial mediators in WAC, and this was partly prevented by WA314 in a YopP-dependent manner (Fig. 3F). The L1 transcripts belong to signal transduction pathways including G-protein coupled receptors, small GTPases and, as in E1 (Fig. 2C), Zinc finger transcription factors (Fig. 3G and H; Fig. S2B through D). L2 transcripts were upregulated by the bacterial mediators, and this was suppressed by T3SS effectors, but YopP, YopM, or YopQ did not play a significant role here (Fig. 3F). The L2 transcripts are enriched in pathways of gene expression, transcription, and MAPK signaling (Fig. 3G; Fig. S2E and F). L3 transcripts were upregulated both by bacterial mediators and the T3SS effectors YopM and YopP and contained Ca^2+^ signaling pathways (Fig. 3F and G; Fig. S2G). L4 transcripts, like the E3 and E4 transcripts, were upregulated by the bacterial mediators, and this was suppressed by WA314 in a YopP-dependent manner (Fig. 3F). The L4 transcripts, like the E3 and E4 transcripts (Fig. 2C), were enriched in central immune signaling pathways, including cellular response to lipopolysaccharide and JAK-STAT signaling that are regulated by NF-κB and interferon pathways (Fig. 3G, I and J; Fig. S2H through K).

Taken together, our data indicate that in human macrophages, inflammatory *Yersinia* components upregulate or downregulate the expression of thousands of genes associated with prominent pathways such as NF-κB- and interferon signaling or the regulation of gene transcription. Other pathways modulated in this context include small GTPases, JAK-STAT signaling, and Ca^2+^ signaling. This extensive inflammatory response is counteracted by the combined activity of the T3SS effectors, of which YopP plays the major but not the sole role.

### YopM and YopQ antagonize YopP in the regulation of gene expression

**Fig 4:**
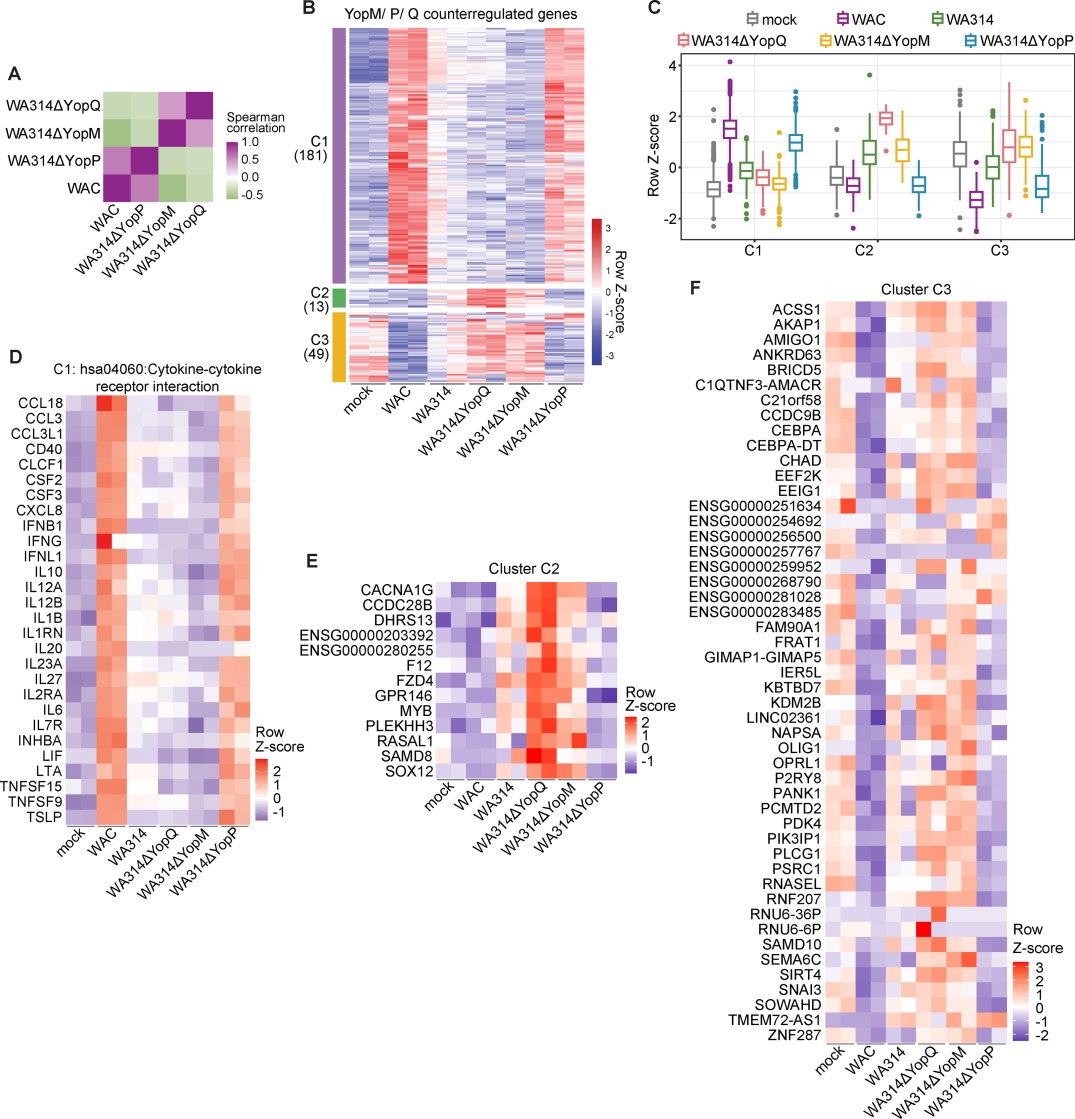
RNA expression profiles and pathways of genes counterregulated by YopP and YopM/YopQ. (**A**) Spearman correlation heatmap representation of gene expression changes between WAC-, WA314ΔYopP-, WA314ΔYopM-, and WA314ΔYopQ-infected macrophages (6 h) for WAC vs WA314 6 h DEGs. (**B**) Clustered heatmap of genes counterregulated by YopM, YopP, and YopQ in comparisons between WAC- and WA314-infected macrophages (6 h). Gene vst counts were row-scaled (row Z-score). Clusters C1–C3 were identified (number of genes in brackets). Two representative replicates are shown, and all replicates are shown in Fig. S3A. (**C**) Boxplots of row-scaled vst counts for genes from clusters C1–C3 (**B**) showing the global expression profile in each cluster when taking into account all replicates. (**D**) Heatmap of row-scaled vst counts for genes enriched in cluster C1 that belong to cytokine-cytokine receptor interaction pathway. Two representative biological replicates are shown, with all replicates depicted in Fig. S3B. (**E**) Heatmap of row-scaled vst counts for genes in cluster C2. Two representative biological replicates are shown, with all replicates depicted in Fig. S3C. (**F**) Heatmap of row-scaled vst counts for genes in cluster C3. Two representative biological replicates are shown, with all replicates depicted in Fig. S3D.

When we analyzed the L1 and L4 clusters in more detail, we noticed that the strain missing YopM and in parts also the strain missing YopQ tended to have opposite effects on gene transcription than the strain missing YopP (Fig. 3F). This phenomenon can also clearly be seen in the genes of the pathways: (i) phospholipase C activating G-protein-coupled receptors, (ii) cellular response to LPS; and (iii) JAK STAT signaling pathways (Fig. 3H through J). To further document the antagonistic activities of YopM and YopQ vs YopP in transcriptional regulation, we performed Spearman correlation analysis of the gene expression changes. The results showed a positive correlation between WAC and WA314ΔYopP and between WA314ΔYopQ and WA314ΔYopM (Fig. 4A). At the same time, WAC and WA314ΔYopP showed a negative correlation versus WA314ΔYopQ and WA314ΔYopM (Fig. 4A). Analysis of counterregulated genes from the RNA-seq data revealed 243 DEGs in three clusters, Counterregulated-1 to Counterregulated-3 (C1–C3), that were affected in opposite directions by YopP vs YopQ and YopM (Fig. 4B and C; Fig. S3A; Table S7). C1 transcripts are inflammatory response genes, including cytokine-cytokine receptor interaction, that are downregulated by YopP. YopM and partly YopQ increased the expression of these genes, thereby counteracting the YopP activity (Fig. 4B, C and D; Fig. S3B). Cluster C2 contains 13 genes that are upregulated by YopP but downregulated by YopQ (Fig. 4B and C). These genes include calcium voltage-gated channel subunit alpha1 G (CACNA1G), Frizzled Class Receptor 4 (FZD4) involved in beta-catenin signaling and RAS protein activator like 1 (RASAL1), the latter has been described in regulating Ras-cyclic AMP pathway (49) (Fig. 4B, C and E; Fig. S3C). C3 transcripts contain genes that are downregulated by the bacterial mediators and upregulated by YopP, and this again is counteracted by YopQ and YopM (Fig. 4B, C and F; Fig. S3D). The C3 transcripts do not belong to a single pathway but include diverse immune signaling receptors, kinases, and transcription factors (Fig. 4F; Fig. S3D). We conclude that part of the YopP effects on gene transcription is systematically counteracted by YopQ and YopM. This counteraction involves key immune signaling pathways of macrophages, and therefore is likely to be of cell biological relevance.

### *Yersinia* effectors suppress phosphorylation of H3S10 at heterochromatin in macrophages

**Fig 5:**
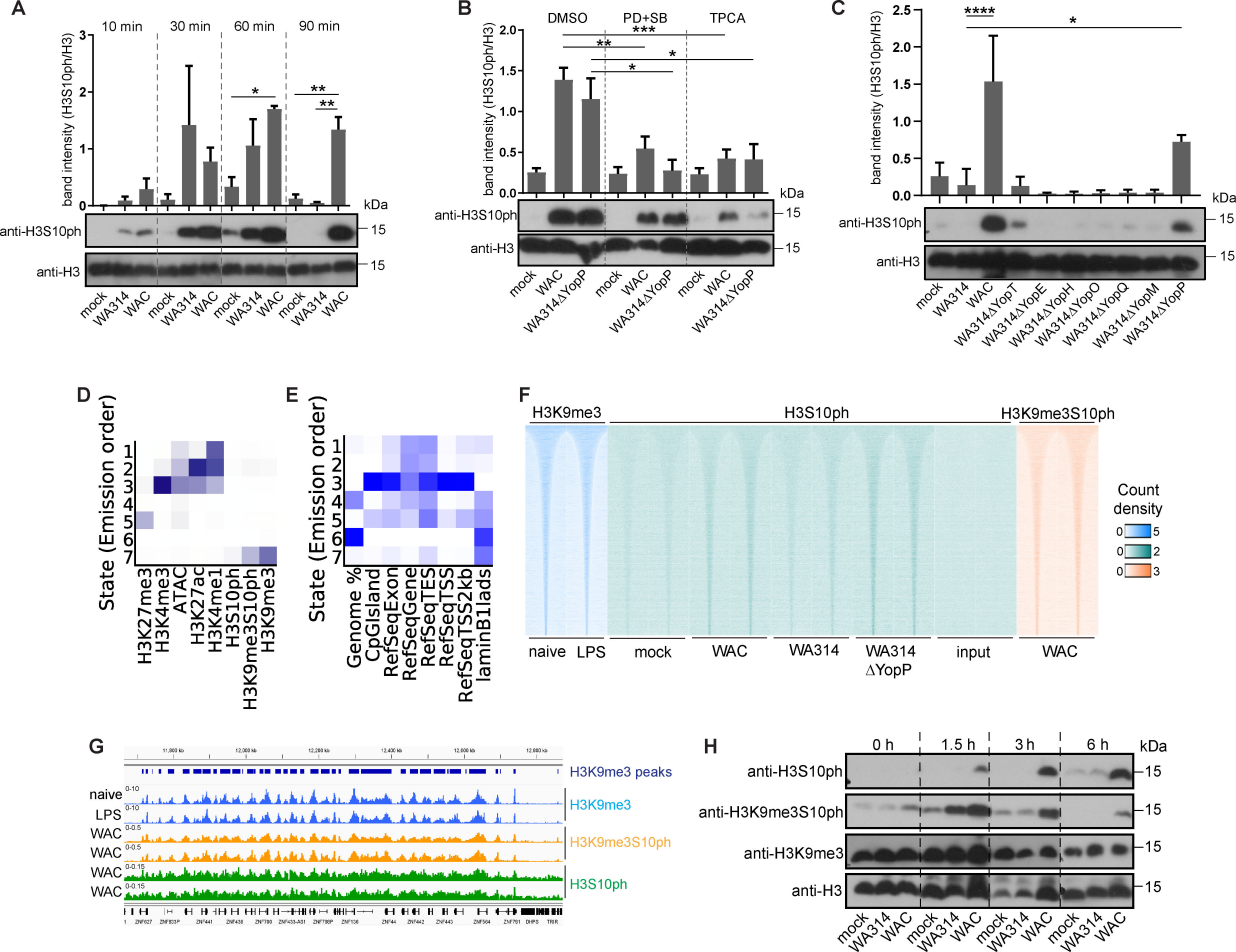
*Yersinia* effectors suppress phosphorylation of H3S10 at heterochromatin regions in human macrophages. (**A**) Bar graph and representative Western blot (bottom) of H3S10ph levels in macrophages that were mock infected or infected with WA314 or WAC for 10 min to 90 min with an MOI of 100. Histone-3 (H3) bands serve as a loading control. Bars show the mean and standard deviation from three independent experiments. *P*-value calculations were performed using one-way ANOVA. *P ≤ 0.05, **P ≤ 0.001. (**B**) Bar graph and representative Western blot (bottom) of H3S10ph levels in macrophages pretreated with solvent (DMSO), MAPK inhibitors (PD + SB), or NF-κB inhibitor (TPCA) for 30–60 min, followed by infection with indicated strains for 3 h with an MOI of 100. H3 bands serve as a loading control. Bars show the mean and standard deviation from six independent experiments. *P*-value calculations were performed using one-way ANOVA. *P ≤ 0.05, **P ≤ 0.001, ***P ≤ 0.001. (**C**) Bar graph and representative Western blot (bottom) of H3S10ph levels in macrophages mock infected or infected with indicated strains for 3 h with an MOI of 100. H3 bands serve as a loading control. Bars show the mean and standard deviation from three independent experiments. *P*-value calculations were performed using one-way ANOVA. *P ≤ 0.05, ****P ≤ 0.0001. (**D**) Heatmap showing ChromHMM analysis assigning different chromatin modifications or open chromatin regions from ATAC-seq (columns) to different chromatin states (1–7; rows). The darker blue color corresponds to a greater probability of observing the mark in the state. The heatmap shows that H310ph, H3K9me3, and H3K9me3S10ph regions are found in state 7 described by closed chromatin (no signal from ATAC-seq). H3S10ph ChIP-seq showed weak enrichment levels. (**E**) Heatmap from ChromHMM analysis showing enrichment of various genomic annotations in different states from (**D**). The analysis shows that state 7 containing H3S10ph, H3K9me3, and H3K9me3S10ph regions (**D**) is enriched for heterochromatin characterized by lamin B1 lamin-associated domains (lads). (**F**) Heatmap showing H3S10ph ChIP-seq signal (green) from macrophages mock infected or infected with the indicated *Yersinia* strains or from input control at H3K9me3 peaks from naïve and LPS-treated macrophages from a publicly available data set (blue) (42). H3K9me3S10ph ChIP-seq from WAC-infected macrophages is depicted in orange. Rows are genomic regions from −10 to + 10 kb around the center of the analyzed regions. *n* = 64,419. (**G**) Peak tracks of ChIP-seq tag densities at H3K9me3 peaks (dark blue bars) for H3K9me3 (blue), H3K9me3S10ph (yellow), and H3S10ph (green) for WAC-infected macrophages and naïve- and LPS-stimulated macrophages from a publicly available data set (42). (**H**) Western blot showing H3S10ph-, H3K9me3S10ph-, and H3K9me3 levels in macrophages mock infected or infected with WA314 or WAC for 0–6 h with an MOI of 100. H3 bands serve as a loading control.

The profound effects of *Yersinia* on macrophage gene expression prompted us to search for overarching regulatory mechanisms potentially underlying this phenomenon. Bacterial inflammatory mediators activate NF-κB and MAP-kinase (MAPK) signaling pathways, which regulate gene expression through a plethora of mechanisms (50, 51). Notably, MAPK signaling has been shown to induce phosphorylation of histone-3 at serine 10 (H3S10ph), an epigenetic modification that primes chromatin for deposition of other modifications or factors that regulate gene expression (52–54). H3S10ph can thereby control gene expression profiles involving hundreds of genes (52, 53). Western Blot analysis revealed that H3S10ph was virtually absent in mock-infected macrophages, whereas both WAC and WA314 induced H3S10ph already after 10 min of infection (Fig. 5A). From 30 to 90 min, WAC-infected cells showed high levels of H3S10ph, whereas in WA314-infected cells, H3S10ph was reduced compared to WAC and even absent after 90 min (Fig. 5A). The suppression of H3S10ph by WA314 was confirmed by two different anti-H3S10ph antibodies (Fig. S4A). This indicates that H3S10ph is induced rapidly by the bacterial mediators and is thereafter suppressed by the T3SS effectors. These kinetics also correlate well with the stimulation of inflammatory gene expression by both WAC and WA314 after 1.5 h of infection and with suppression of gene expression by WA314 after 6 h of infection (Fig. 2 and 3). Pretreatment with MAPK inhibitors PD98059 and SB203580 (PD + SB) and an NF-κB inhibitor (TPCA) suppressed H3S10ph after WAC infection, indicating that H3S10ph is induced through MAPK- and NF-κB-mediated pathways (Fig. 5B). H3S10ph modification also associates with the cell cycle, where H3S10ph occupancy along the entire length of chromosomes is observed during mitosis (55). However, primary human macrophages are terminally differentiated and do not undergo cell division, which indicates that the changes in H3S10ph levels during *Yersinia* infection take place during interphase and are not due to the regulation of cell cycle (56).

To investigate which of the effectors may suppress H3S10ph, the macrophages were infected with strains lacking each of the effectors individually (Fig. 5C). *Yersinia* mutants lacking YopT, -E, -H, -O, -Q, or -M suppressed H3S10ph to the same degree as WA314, indicating that none of these effectors alone is capable of inhibiting H3S10ph (Fig. 5C). In comparison, WA314ΔYopP inhibited H3S10ph about half as effectively as WA314 (Fig. 5C). This suggests that YopP plays the major role in suppressing H3S10ph and that the remaining inhibition of H3S10ph is due to a combined effect of the other Yops. Treatment with the MAPK- and NF-κB inhibitors, when compared to DMSO control, reduced H3S10ph in WA314ΔYopP-infected cells, consistent with the idea that YopP inhibits H3S10ph through inhibition of MAPK and NF-κB signaling (Fig. 5B).

In the next step, we performed H3S10ph chromatin immunoprecipitation and sequencing (ChIP-seq) to analyze at which chromatin regions H3S10ph occurs. The macrophages were mock-infected or infected with WAC, WA314, or WA314ΔYopP for 3 h, followed by the H3S10ph ChIP-seq (Materials and Methods). We performed chromatin state discovery and characterization with ChromHMM analysis (57, 58) to test whether H3S10ph is localized at active/open chromatin and may directly associate with the expression of inflammatory genes or inactive/closed chromatin states. For this, we integrated our own data set from H3K4me3, H3K27me3, H3K4me1, and H3K27ac ChIP-seq in *Yersinia*-infected primary human macrophages (28) and publicly available data sets from H3K9me3 ChIP-seq and ATAC-seq in primary human macrophages (42). H3S10ph showed weak enrichment, but notably, was detected in regions marked by repressive heterochromatin mark H3K9me3 (Fig. 5D, F and G). The H3K9me3 mark is found in lamin B1 lamin-associated domains (LADs) that signify inactive and closed chromatin (Fig. 5E). H3S10ph was not associated with active chromatin regions containing modifications associated with active gene transcription such as H3K4me3 and H3K27ac, enhancer regions marked with H3K4me1 or open chromatin regions from ATAC-seq data (Fig. 5D and E). We next employed an antibody that exclusively recognizes the double chromatin modification H3K9me3/H3S10ph but not the individual H3K9me3 and H3S10ph modifications. Western blot analysis revealed that the combined H3K9me3/H3S10ph modification increased in parallel to the H3S10ph modification in WAC-treated cells and was strongly inhibited, like the H3S10ph modification, in the WA314-treated cells from 3 to 6 h of infection (Fig. 5H). In contrast, the H3K9me3 mark alone did not show these kinetics (Fig. 5H). ChIP-seq analysis using the H3K9me3/H3S10ph antibody in the WAC-infected macrophages showed a better enrichment in the H3K9me3-marked regions than when the H3S10ph antibody was used (Fig. 5D, F and G). In WAC-infected macrophages, 97% of the H3K9me3S10ph peaks were found at introns and distal intergenic regions, while only 0.5% of the peaks were associated with the promoter regions (Fig. S4B). H3K9me3S10ph was not associated with enhancer regions marked by H3K4me1 (Fig. 5D; Fig. S4C), and H3K9me3S10ph was observed over large domains at chromatin similar to H3K9me3 distribution (Fig. S4D). Approximately 40%–50% of both upregulated and downregulated genes by WAC vs mock associated with H3K9me3S10ph peaks, indicating no direct association with gene expression (Fig. S4E). We conclude that the massive initiation of gene transcription by the *Yersinia* mediators is associated with a strongly increased deposition of H3S10ph near the repressive chromatin mark H3K9me3, which may prepare/prime inactive chromatin regions for access by transcription-promoting factors (59) and affect macrophage chromatin organization. Conversely, inhibition of gene transcription by YopP and the cooperative action of the remaining Yops are associated with a significantly reduced amount of H3S10ph at the repressive chromatin mark, which most likely inhibits chromatin processes associated with rapid induction of gene transcription in the inflammatory response on a global level.

### *Yersinia* effectors YopQ and YopP cooperate to downregulate inflammasome formation in human macrophages

**Fig 6:**
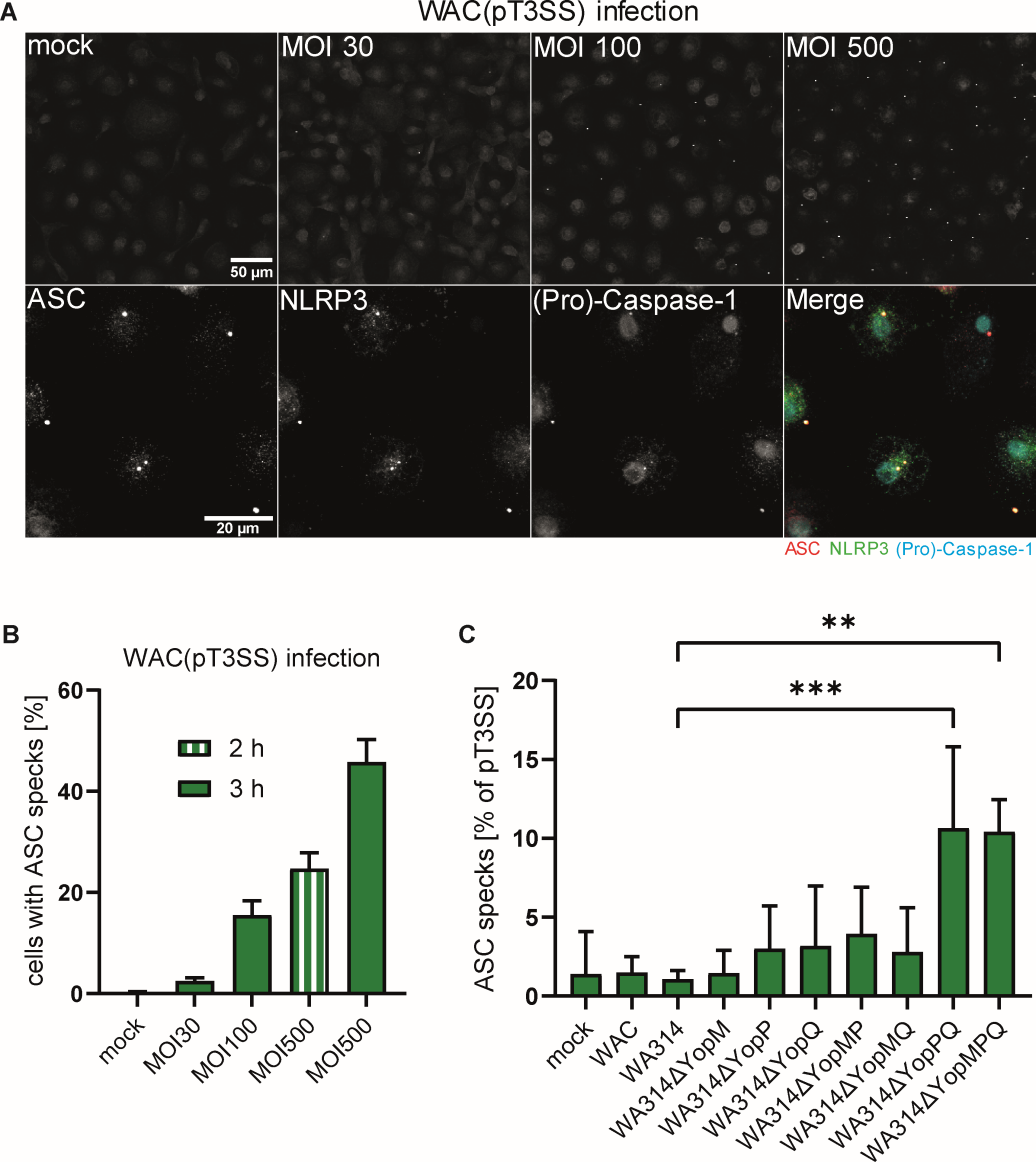
*Yersinia* effectors cooperate to downregulate inflammasome formation in human macrophages. (**A**) (Upper row) Human macrophages form inflammasomes upon infection with a *Yersinia* strain that overproduces translocation pores. Macrophages were mock-infected or infected with indicated MOIs of strain WAC(pT3SS) for 3 h and immunostained for endogenous ASC. (Bottom row) NLRP3-eGFP-transfected human macrophages were infected with WAC(pT3SS) at an MOI of 500 for 2 h and immunostained for endogenous ASC and (Pro)-Caspase-1. Merge shows overlay of ASC (red), NLRP3 (green), and (Pro)-Caspase-1 (blue). (**B**) Dose and time dependency of ASC speck formation in human macrophages infected with indicated MOIs of WAC(pT3SS) for 3 h or 2 h. The 2 h value with MOI 500 (striped bar) gives a sufficiently high percentage of ASC-containing cells with a still low number of pyroptotic macrophages, and the values in (**C**) were normalized to this value. Bars represent mean and standard deviation from at least two biological replicates with 600 cells counted per replicate. (**C**) Effect of individual or combined *Yersinia* effector mutants on NLRP3 inflammasome formation in human macrophages. Macrophages were mock-infected or infected with the indicated strains at an MOI of 500 for 2 h. Bars represent the mean and standard deviation of at least two replicates with 600 cells counted per replicate. The number of ASC specks formed in macrophages from each donor after infection, with WAC(pT3SS) (MOI 500, 2 h; from panel B) was used as a reference value. ASC formation values of the *Yersinia* mutants in the macrophages were normalized to the corresponding reference value. Significance was calculated using an ordinary one-way ANOVA. *P-value ≤ 0.05, **P ≤ 0.001, ***P ≤ 0.001

It has been reported that YopQ/YopK suppresses the activation of the NLRP3 inflammasome induced by the T3SS translocation pores and that YopM inhibits the activation of the pyrin inflammasome induced by the inactivation of Rho-GTP-binding proteins (33, 36, 60, 61). These results were almost exclusively obtained in bone marrow-derived mouse macrophages, and no data on the individual or combined effects of *Yersinia* T3SS effectors on canonical inflammasomes in primary human macrophages have been reported. Interestingly, in human macrophages, activation of the non-canonical caspase-4 inflammasome by *Y. pseudotuberculosis* was synergistically inhibited by the T3SS effectors YopE, YopH, and YopK (40). To investigate the formation of canonical inflammasomes in primary human macrophages, we infected the cells with strain WAC(pT3SS), a WAC strain harboring and overproducing the T3SS, including translocation pores but lacking the Yop effectors (62). WAC(pT3SS) infection produced time- and dose-dependent ASC specks in the macrophages (Fig. 6A and B). These specks contain NLRP3-GFP and Caspase-1 and therefore qualify as clusters of inflammasomes (Fig. 6A) (63). Macrophages not infected (mock) or infected with avirulent WAC, wild-type WA314, or with wild-type strains containing single deletions of YopM, YopP, or YopQ showed only a minimal ASC speck formation (Fig. 6C). Altogether, these data show that (i) the *Yersinia* T3SS structural components are strong activators of inflammasome formation, (ii) *Yersinia* inflammatory mediators alone (like LPS) do not cause formation of inflammasomes, (iii) WA314 through its Yops completely blocks inflammasome formation triggered by the T3SS components, and (iv) the removal of YopQ, YopM, or YopP alone is not sufficient to reverse the blockage of inflammasome formation. To find out whether Yops cooperate to inhibit inflammasome formation, we infected the macrophages with the double mutants lacking YopM/YopP, YopM/YopQ, and YopP/YopQ and the triple mutant lacking YopM/YopP/YopQ. The YopP/YopQ and YopM/YopP/YopQ mutants showed significant and equal increases in inflammasome formation, whereas the other mutants had no effect (Fig. 6C). The effects on inflammasome formation were not due to different numbers of cell-associated bacteria for the different strains (Fig. S5C). To be able to identify even small differences in ASC speck/inflammasome formation between the different bacterial strains in these experiments, we employed a multiplicity of infection (MOI) of 500 but with a reduced infection time of 2 h (Fig. 6B). Even though this appears to be a putatively unphysiological MOI, cells did not show signs of stress or changes in morphology under these conditions (Fig. 6A).

That YopM had no effect on inflammasome formation in primary human macrophages, either alone or in combination with YopQ and/or YopP, is in contrast to mouse macrophages, in which YopM plays an important role in blocking the pyrin inflammasome (60). Pyrin is expressed in primary human macrophages (64), and we also observed significant upregulation of pyrin gene *MEFV* after 6 h of infection by both avirulent WAC and virulent WA314 Yersinia strains vs mock, by 64-fold and 44-fold, respectively (Table S3). Inflammasome activation by a *Y. pseudotuberculosis* YopK (homolog of YopQ) mutant in murine macrophages has been proposed to be due to hypertranslocation of the translocation pore components YopB/YopD (37). We recently showed that most of the translocated YopD and YopB are integrated into phagosomal membranes and form pores there (65, 66), and pore formation occurred shortly before phagosomal rupture and activation of non-canonical inflammasomes (67). We therefore quantified total and pore-localized YopD in macrophages infected with wild-type and YopQ deletion mutant but did not observe any differences (Fig. S4F). Thus, the level of YopD is not elevated in the human primary macrophages infected with the *Y. enterocolitica* YopQ deletion mutant vs. the wild type. Overall, these data suggest that YopP and YopQ must work together to inhibit inflammasome formation in primary human macrophages.

### *Yersinia* effector YopH suppresses Ca^2+^ spikes in human macrophages

**Fig 7:**
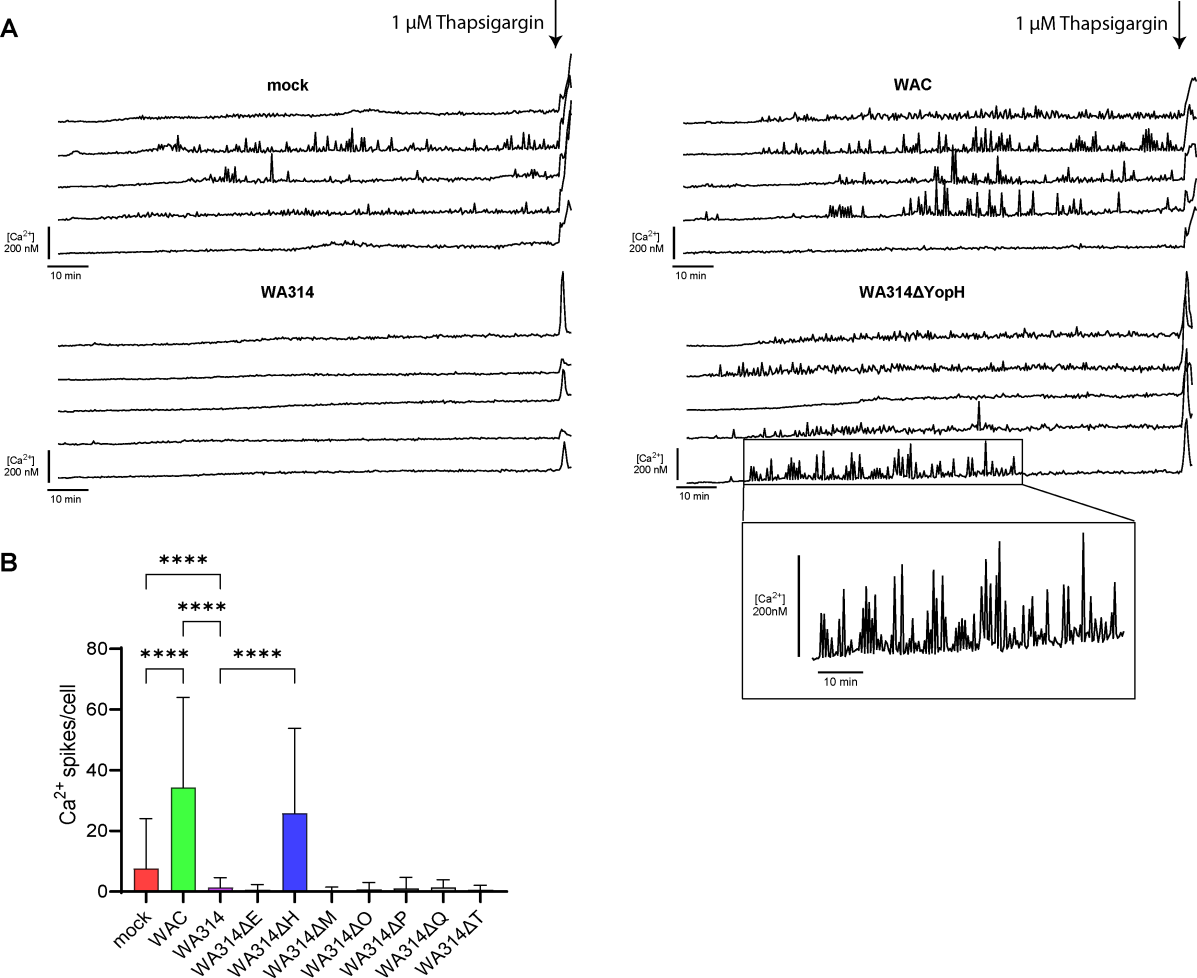
*Yersinia* effector YopH blocks rises in intracellular Ca^2+^ concentration in human macrophages. (**A**) Five representative single-cell Ca^2+^ concentration changes in macrophages mock-infected or infected with each of the indicated *Yersinia* strains with an MOI of 50 for 2 h and loaded with Cal520-AM and FuraRed-AM. Cells were imaged with three frames per minute for 2 h. Stimulation with 1 µM Thapsigargin at 2 h post-infection (hpi) was used as a positive control. (**B**) Quantification of Ca^2+^ spikes/cell accumulated until 2 hpi. Ca^2+^ spikes were defined as ΔF_em1_ > 20% (Materials and Methods). Data represent mean and standard deviation of three independent experiments with altogether 238–673 cells evaluated for each condition. For statistical analysis, ordinary one-way ANOVA with multiple comparisons was used. ****P ≤ 0.0001.

Changes in the intracellular Ca^2+^ concentration are a universal regulator of macrophage immune activities, including gene expression and cytokine production (41). Although *Y. pseudotuberculosis* has been described to affect intracellular Ca^2+^ fluctuations via the effector YopH in human neutrophils (16, 68), there are no reports on how Ca^2+^ responses are altered by pathogenic *Y. enterocolitica* in macrophages. Ca^2+^ imaging of mock-infected macrophages revealed that within a period of 120 min Ca^2+^ fluctuations accumulated to 7.6 Ca^2+^ spikes/cell with a mean increase of 54 nM (Fig. 7A and B; Fig. S5A and B). In WAC-infected cells, the number and mean increase in Ca^2+^ spikes were significantly elevated compared to mock (34 Ca^2+^ spikes/cell; increase: 157 nM) (Fig. 7A and B; Fig. S5B). This suggests the stimulation of Ca^2+^ signaling by LPS or other activators such as Invasin. WA314-infected cells showed much lower number and mean increase in Ca^2+^ spikes (1.4 Ca^2+^ spikes/cell; increase: 36.4 nM), suggesting that one or more effectors suppress both baseline and *Yersinia* mediator induced Ca^2+^ fluctuations (Fig. 7A and B; Fig. S5A and B). Infection of the macrophages with single mutants of each of the seven Yops showed that only infection with the YopH-deficient strain resulted in Ca^2+^ spikes that were similar in number and increase to the spikes in WAC-infected cells (25.9 Ca^2+^ spikes/cell; increase: 66.8 nM) (Fig. 7A and B; Fig. S5B; Movie S1). We conclude that baseline and *Yersinia*-stimulated Ca^2+^ signaling in human macrophages is specifically and selectively abolished by YopH.

## DISCUSSION

The strategic cooperation between bacterial virulence factors was referred to as a stratagem several decades ago (69, 70). Meanwhile, numerous reports indicate that the activities of the different effectors of a pathogen can be linked in a variety of ways (71). A recent article investigated the network of T3SS effectors of *Citrobacter rodentium* in mouse pathogenicity (4). One mutant strain that lacked 19 of the 31 *C*. *rodentium* effectors was still virulent, indicating considerable redundancy between the effectors in this pathogen. The remaining 12 effectors formed a robust network and could not be further contracted without compromising virulence (4). In comparison, pathogenic *Y. enterocolitica* spp. contain only seven effectors, and infection of mice suggests that each of the five effectors—YopP, YopQ, YopM, YopE, and YopH—is essential for the maintenance of full pathogenicity (72). Thus, most of the *Yersinia* effectors already fulfill unique and non-redundant functions during infection. Most studies investigating Yersinia T3SS effectors have focused on the activity of individual effectors in transformed cell lines or mouse macrophages. To obtain more detailed information on how key immune functions of human macrophages are jointly altered by the *Yersinia* effectors, we here tested their individual and especially combined effects on the expression of inflammatory genes, the activation of inflammasomes, and changes in intracellular Ca^2+^ concentration.

YopP had the most profound effect on the inflammatory-mediator-induced changes in gene transcription. It suppressed the upregulation or downregulation of altogether >2,500 genes, most certainly by virtue of its inhibition of NF-κB and MAP-kinase signaling. As a potential higher-level regulatory mechanism of gene transcription in infected macrophages, we investigated the level of phosphorylated serine-10 in histone-3 (H3S10ph), which was shown to be induced by LPS in mouse macrophages (73) and is efficiently subverted by pathogenic bacteria *Listeria monocytogenes*, *Streptococcus pneumoniae,* and *Pseudomonas aeruginosa* (74–76). The *Yersinia* inflammatory mediators induced an NF-κB- and MAP-kinase-dependent increase, followed by a YopP-mediated decrease of H3S10ph. H3S10ph colocalized with the repressive histone mark H3K9me3 in the macrophage genome, suggesting that it regulates the accessibility of genes to transcriptional activators (53, 77). Phosphorylation of H3S10 at H3K9me3 is a mechanism known during mitosis as “phospho-methyl switch” regulating HP1 dissociation and cell division (78, 79), while phosphorylation of H3S10 at H3K9me2 regulates gene expression upon growth factor stimulation (59). In *Yersinia*-infected macrophages, H3S10ph was found at distal intergenic and intronic regions, and there was no direct link between H3S10ph deposition and upregulation or downregulation of specific genes. This is in contrast to H3 lysine 4 trimethylation (H3K4me3) and H3 lysine 27 acetylation (H3K27ac) modifications, which showed clear positive correlation with associated gene induction (28). Therefore, in *Yersinia*-infected primary human macrophages, H3S10ph may be involved in chromatin organization and rapid induction of transcriptional response by inflammatory mediators of bacteria via MAPK and NF-κB pathways. Inhibition of H3S10ph by YopP contributes to its overwhelming negative effect on gene transcription and inflammatory response.

Surprisingly, we found that YopM and partly also YopQ counteracted selected effects of YopP on gene transcription, for example, of cytokine signaling pathways. Previous studies have already shown that YopM can reverse the YopP-mediated downregulation of IL-10 production. To do so, YopM caused the activation of RSK kinases in the host cell nucleus and nuclear translocation of the transcription factor STAT3 (22, 23). The additional >200 genes identified here as oppositely regulated between YopP and YopM/YopQ include not only a large number of cytokines but also components of other inflammatory signaling pathways. How the opposing regulation of these genes eventually contributes to the pathogenicity of the bacterium remains unclear at the moment, but it can be speculated that YopM reverses exactly those activities of YopP that would otherwise hinder the infection progression. For example, reversal of the YopP-inhibited expression of the immunosuppressive cytokine IL-10 and JAK-STAT signaling genes may be beneficial for the pathogen (22, 80). Furthermore, YopM may balance the expression of cell survival and inflammatory genes which are suppressed by YopP/J, thus regulating the induction of caspase-8-dependent cell death that is beneficial for the host (81, 82). Another finding of our study is that although YopQ by itself had no major influence on gene expression, a group of signaling genes whose products are involved in Ca^2+^ -, beta-catenin-, and RAS signaling are downregulated by YopQ. This result could be a first step toward the elucidation of the molecular mode of action of YopQ.

When we investigated the suppression of canonical inflammasome/speck formation by the *Y. enterocolitica* effectors, we found that single knockouts of each of the three effectors—YopM, YopQ, and YopP—had no effect in human macrophages. Only after infection with the combined YopP/YopQ-deficient strains, the suppression of speck formation significantly reduced. We conclude that, as opposed to the situation in mouse macrophages, YopM, neither alone nor in combination with YopP or YopQ, affected inflammasome/speck formation in human macrophages. This may be due to the fact that in human macrophages, the pyrin inflammasome response, which can be inhibited by YopM, requires prolonged priming with either LPS or type I and type II interferons and an increase in pyrin protein levels (64). Our RNA-seq data show that the pyrin gene expression was significantly upregulated by both virulent and avirulent *Yersinia* strains in the macrophages, but only after 6 h of infection. Therefore, the lack of a pyrin inflammatory response could be the reason why YopM is dispensable for inhibiting speck formation under our experimental conditions. That a pyrin inflammasome, which senses bacterial effector-mediated inactivation of Rho and Rac, is not required in human macrophages may potentially also be explained by the production of alternative Rho-GTPases in these cells upon infection with *Y. enterocolitica* (28). The newly produced Rho GTP-binding proteins may be insensitive to YopE and YopT and could take over their function. Interestingly, it has been reported that the pryin inflammatory response is also not activated by numerous other Rho GTPase-inhibiting toxins in primary human macrophages (64). Two other mechanisms through which YopM potentially could inhibit inflammasome activation have been reported. First, YopM directly associates with the dead-box helicase DDX3 (23), which has been shown to bind to NLRP3 and drive inflammasome activation in human macrophages (83). Second, YopM has been proposed to directly bind caspase-1 and thus block formation of the mature inflammasome (84). However, none of the mechanisms seems to play a role in our system.

In murine macrophages, *Y. pseudotuberculosis* YopK (homolog of YopQ) mutants cause hypertranslocation of YopB/D, which causes Caspase-11 activation followed by non-canonical NLRP3 activation (37). However, we did not find tha*t the Y. enterocolitica* YopQ mutant causes enhanced speck formation in primary human macrophages. We therefore quantified the amount of YopB/D in wild-type- and YopQ mutant-infected macrophages but did not observe any differences. This underscores the differences in the response to bacterial T3SS activity and inflammasome regulation between murine and human macrophages. The result that a combination of YopP and YopQ was required to inhibit inflammasome/speck formation may be explained by the fact that in the human macrophages, LPS-triggered expression of inflammasome constituents and NLRP3-mediated inflammasome activation must be inhibited in parallel by YopP and YopQ, respectively (85). Future studies should investigate these intriguing possibilities. Finally, as was shown before in neutrophils, calcium signaling in the form of repeated fluctuations of intracellular Ca^2+^ concentration in the *Yersinia*-infected macrophages was blocked completely by YopH, and none of the other effectors had any effect on it.

In summary, we propose that Yersinia effectors act antagonistically, cooperatively, and individually on key immunoregulatory pathways in human macrophages to ultimately suppress the activation state of these immune cells.

## MATERIALS AND METHODS

### Bacterial strains

*Yersinia enterocolitica* strains used in this study are derivatives of the serotype O:8 strain WA314 harboring the virulence plasmid pYVO8 (62). WAC is the plasmidless derivative of WA314. WAC(pT3SS) is a derivative of WAC, which harbors the pT3SS plasmid encoding for the type three secretion system (T3SS). WA314ΔYopM, WA314ΔYopQ, WA314ΔYopH, WA314ΔYopO, WA314ΔYopT, WA314ΔYopD, and WA314ΔYopE were constructed by replacing the corresponding gene in WA314 with a kanamycin resistance cassette (72). WA314ΔYopP was generated by insertional inactivation of the yopP gene (86). WA314ΔYopMP and WA314ΔYopPQ are derivatives of *Yersinia enterocolitica* WA314. WA314ΔYopMQ and WA314 ΔYopMPQ were generated using a CRISPR-Cas12a-assisted recombineering approach as described previously (67, 87). Table S1 provides an overview of the bacterial strains used in this study.

### Cell culture

Human peripheral blood monocytes were isolated from buffy coats as described previously (88). Cells were cultured in monocyte medium (RPMI1640, 20% autologous serum, 1% penicillin/streptomycin) at 37°C and 5% CO_2_. The medium was changed every 3 days until cells were differentiated into macrophages on day 7 after isolation. Macrophages were used for infection 1 week after the isolation, except for RNA-seq samples from batch 1 in Table S2 for which cells were used after 2 weeks of isolation. Gene expression profiles were not affected by the time after isolation (28).

### Infection of cells

On the day before infection of primary human macrophages, the cell medium was changed to RPMI1640 without antibiotics, and serum and precultures of *Y. enterocolitica* strains (Table S1) were grown overnight in LB medium with appropriate antibiotics at 27°C and 200 rpm. On the day of infection, precultures were diluted 1:20 in fresh LB medium without antibiotics and incubated for 90 min at 37°C and 200 rpm to induce the activation of the *Yersinia* T3SS machinery and Yop expression. Afterwards, the bacteria were pelleted by centrifugation for 10 min at 6,000 × *g*, 4°C, and resuspended in 1 mL ice-cold phosphate-buffered saline (PBS) containing 1 mM MgCl_2_ and CaCl_2_. The optical density OD_600_ was adjusted to 3.6, and afterwards, macrophages were infected at MOI from 50 to 500 as indicated in the figure legends. Cell culture plates were centrifuged for 2 min at RT and 200 × *g* to sediment bacteria on the cells and synchronize infection. Cells were incubated at 37°C for time specified in the figure legends. For experiments using inhibitors for the NF-κB pathway (TPCA, Cell Signaling) and MAPK pathway (PD98059, Cell Signaling & SB203580, Cayman Chemical), inhibitors were added 30–60 min prior to infection at a final concentration of 10 µM.

To ensure equal bacterial load during infections, all used strains were tested for their colony-forming units (cfu). For the assay, 2 × 10^5^ cells per well were seeded. After infection, cells were washed and lysed with 0.5% Digitonin in PBS at the indicated timepoints. Serial dilutions of the lysates were plated on LB agar plates. Colonies of appropriate dilutions were counted after growth at 27°C for 48 h, and the cfu/well was calculated (Fig. S5C).

### Histone extraction

Histones were extracted from primary human macrophages following a modified acid extraction protocol (89). 0.5–1.5 × 10^6^ primary human macrophages were washed with ice-cold PBS and harvested in 1 mL PBS. Cells were pelleted by centrifugation at 700 × *g* for 5 min at 4°C, and the supernatant was discarded. Lysis of the cells was done by incubation of the pellet in 0.5 mL Hypotonic lysis buffer (10 mM Tris-HCl, pH 8.0, 1 mM KCl, 1 mM DTT, 1.5 mM MgCl_2_, 1× Complete Protease Inhibitor, 1× PhosStop Phosphatase Inhibitor), rotating at 4°C for 60–90 min and controlled by microscopy. Isolated nuclei were pelleted by centrifugation at 10,000 × *g* for 10 min at 4°C, supernatant was discarded, and the pellet was resuspended in 0.2 M HCl and lysed by incubating at 4°C rotating overnight. Nuclear debris was removed by centrifugation at 16,000 × *g* for 10 min at 4°C, and the supernatant was transferred to a new tube. Histones were precipitated using incubation with trichloroacetic acid (TCA) (final concentration 33%) for 30 min on ice. Pelleting of precipitated histones was carried out by centrifugation at 16,000 × g for 10 min at 4°C, and the supernatant was discarded. The pellet was washed twice with 500 µL ice-cold acetone, centrifuged in between at 16,000 × *g* for 5 min at 4°C, finally air-dried, and dissolved in 100 µL millipore water. Concentration of the dissolved histones was determined using Bradford protein assay, and the quality and purity of the extracted histones were controlled by SDS-PAGE and subsequent Coomassie staining of the gel.

### Western blot analysis

Equal amounts of histone proteins were separated by SDS-PAGE and transferred to polyvinylidene difluoride membrane (Immobilon-P, Millipore, Schwalbach, Germany) by semi-dry blotting. The membrane was incubated with 3% bovine serum albumin (BSA) (wt/vol) in TBS supplemented with 0.05% Tween 20 (TBS-T) for 30–60 min and subsequently with primary antibodies at 4°C overnight. Primary antibodies used in this study were rabbit anti-Histone H3 antibody (Cell Signaling, 1:3,000 diluted), rabbit anti-Histone H3S10ph (Invitrogen and Abcam, 1:1,000 diluted), rabbit anti-Histone H3K9me3 (Abcam, 1:1,000 diluted), and rabbit anti-Histone H3K9me3S10ph (Abcam, 1:1,000 diluted). The membrane was washed thrice with TBS-T and afterward incubated at room temperature for 1 h with donkey anti-rabbit IgG (Cell Signaling, GE Healthcare) as a secondary antibody in a dilution of 1:10,000–1:25,000. Membrane was washed thrice with TBS-T, and antibody signals were visualized with chemiluminescence technology (Supersignal West Femto, Pierce Chemical, Rockford, USA) and captured on X-ray films (Fujifilm, Düsseldorf, Germany). Developed films were scanned (CanonScan 4400 F, Canon, Tokyo, Japan) to quantify protein band intensity. Quantification of signal intensity of scanned films was performed using ImageJ analysis software Version 1.53 (National Institute of Health, NIH).

### Immunofluorescence staining and confocal microscopy

Primary human macrophages were washed once with PBS and detached with Accutase (Thermo Fisher Scientific, Waltham, USA) for 30 min at 37°C. Cells were scraped off, mixed with an equal volume of RPMI1640 medium (Gibco, Carlsbad, USA), counted, and seeded at a density of 6 × 10^4^ cells onto glass coverslips (Marienfeld GmbH, Lauda-Königshafen, Germany). After 30 min at 37°C, the medium was changed to monocyte medium (RPMI1640, 20% autologous human serum, 1% penicillin/streptomycin) and cells were incubated overnight at 37°C. Infection was done as described above. After infection, cells on coverslips were washed once with PBS, fixed with 4% PFA in PBS for 7 min, and washed twice with PBS.

For YopD fluorescence intensity assessment, the staining was performed as described previously (65). Briefly, fixed cells after infection were permeabilized (0.1% Triton X-100/PBS) for 15 min at RT and blocked (PBS, 3% [wt/vol] BSA) for 1 h at RT. The translocon protein YopD was stained using a primary anti-YopD antibody (1:50 in PBS, 3% [wt/vol] BSA), washed thrice, and a secondary anti-rabbit antibody coupled to AlexaFluor 568 (1:200 in PBS, 3% [wt/vol] BSA; Invitrogen). Fluorophore-coupled phalloidin and DAPI were added to the staining solution.

For the IF staining of inflammasome components, cells were permeabilized (PBS, 0.1% ([wt/vol) Triton X-100) for 15 min at RT immediately after fixation, then washed twice and blocked with blocking solution (PBS, 3% [wt/vol] BSA, 0.05% Triton X-100) for 30–60 min at RT in a humid chamber. Next, coverslips were transferred into blocking solution containing the desired primary antibodies (mouse anti-ASC, Santa Cruz, 1:50; rabbit anti-Caspase-1, Invitrogen, 1:100) and incubated overnight at 4°C. Washing with PBS was done thrice, and incubation with secondary antibodies, DAPI, and Phalloidin coupled to Alexa Fluor dyes (1:200 in PBS, 3% [wt/vol] BSA; Invitrogen) was done for 60 min at RT in a humid chamber.

For all stainings, coverslips were washed thrice and finally mounted in Prolong Glass Antifade Mountant on an object slide. Images were acquired using the laser scanning microscope Olympus FV3000 with a 20× air objective (NA 0.8) or the 60× oil objective (NA 1.4) and the Olympus FV3000 Software (Evident Europe GmbH, Germany).

### Ca^2+^ imaging of primary human macrophages during *Yersinia enterocolitica* infection

Primary human macrophages were differentiated as described above. For Ca^2+^ imaging, cells were seeded in eight-well chamber slides (ibidi) and loaded with Cal520-AM (5 µM), FuraRed-AM (10 µM), and Pluronic F127 (0.05%) in RPMI for 60 min at 37°C. Cells were washed and maintained in Ca^2+^-measuring buffer (NaCl 140 mM, KCl 5 mM, MgSO_4_ 1 mM, CaCl_2_ 1 mM, NaH_2_PO4 1 mM, Glucose 5.5 mM, HEPES 20 mM, pH 7.4) (90). Imaging was performed with the spinning disk microscope Visitron SD-TIRF (Nikon Eclipse TiE, Nikon) with a 20× CFI Plan Fluor DLL Phase objective (NA 0.51) and a sCMOS camera (Photometrics Prime 95B) in an incubation unit at 37°C and 5.0% CO_2_. Acquisition of images was carried out using the VisiView v4 software (Visitron Systems). The following settings were used for imaging Ca^2+^ signals: excitation (ex): 488 nm; emission1 (em1): 525/50 nm; em2: 700/75 nm; exposure time: 150 ms; acquisition rate: three frames per minute. Bacteria were prepared as described above and kept in Ca^2+^-measuring buffer. Cells were infected with an MOI of 50, 3 min after the start of acquisition to capture baseline fluorescence. Imaging was conducted continuously for a period of 120 min after infection (91). Cells were stimulated with 1 µM Thapsigargin as a positive control 120 min following infection. Calibration of R_max_ (10 µM Ionomycin) and R_min_ (10 µM Ionomycin and 10 mM EGTA) was performed after each experiment (90). Images were background and motion corrected (92). Single cells were defined as regions of interest. Image processing was performed using Fiji (93). Intracellular Ca^2+^ concentration was calculated with the *K*d of Cal520-AM (*K*d = 320 nM) (94). Ca^2+^ spikes were defined as an increase of ΔF_em1_ > 20%, which exceeded the standard deviation of mock-infected macrophages by more than threefold (95). Quantification of fluorescence intensity was performed using Microsoft Excel, MATLAB R2023b (MathWorks), and with the assistance of ChatGPT (OpenAI).

### Statistical analysis

Statistical analysis was performed using GraphPad Prism version 10.0. At least three independent experiments were compared by paired t-test, one-way ANOVA with Bonferroni’s post-test, or two-way ANOVA if not indicated otherwise. *P*-values ≤0.05 were considered statistically significant.

### RNA-seq

Total RNA of 1-2 × 10^6^ human macrophages (for number of biological replicates/macrophage donors per condition, see Table S2) was isolated using the RNeasy extraction kit (Qiagen), including DNase treatment according to the manufacturer’s instructions. RNA integrity of the isolated RNA was analyzed with the RNA 6000 Nano Chip (Agilent Technologies) on an Agilent 2100 Bioanalyzer (Agilent Technologies). mRNA was extracted using the NEBNext Poly(A) mRNA Magnetic Isolation module (New England Biolabs), and RNA-seq libraries were generated using the NEBNext Ultra RNA Library Prep Kit for Illumina (New England Biolabs) as per the manufacturer’s recommendations. Concentrations of all samples were measured with a Qubit 2.0 Fluorometer (Thermo Fisher Scientific), and the fragment length distribution of the final libraries was analyzed with the DNA High Sensitivity Chip (Agilent Technologies) on an Agilent 2100 Bioanalyzer (Agilent Technologies). All samples were normalized to 2 nM and pooled equimolar. The library pool was sequenced on the NextSeq500 (Illumina) with 1 × 75 bp for batch 2 samples, 1 × 50 bp for batch 1 samples, and 1 × 75 bp for batch 3 samples (Table S2).

### RNA-seq data

Part of the RNA-seq data were obtained from already publicly available sources: ArrayExpress database at EMBL-EBI (www.ebi.ac.uk/arrayexpress) under accession number E-MTAB-10473 and European Nucleotide Archive (ENA) at http://www.ebi.ac.uk/ena/data/view/PRJEB10086. RNA-seq data for the first time used in this study have been deposited in the ArrayExpress database at EMBL-EBI (www.ebi.ac.uk/arrayexpress) under accession numbers E-MTAB-10602 and E-MTAB-15035. For a detailed description of sample sources and batches used in this study, refer to Table S2.

### RNA-seq analysis

Raw FASTQ files from RNA sequencing were processed using the nf-core/rnaseq pipeline (v1.3) (https://nf-co.re/rnaseq/) within the nf-core framework (96). The analysis was performed with Nextflow (v21.04.0) (97), utilizing STAR (v2.6.1d) (98) for alignment to the GRCh38 human genome. Gene-level quantification was carried out using featureCounts (v2.0.1) (99), which counts reads mapped to each gene. Transcript-level counts were imported using tximport (v1.30.0) (100) package and summarized to gene-level counts with EnsDb.Hsapiens.v86 package (DOI: 10.18129/B9.bioc.EnsDb.Hsapiens.v86) and used for differential expression analysis with DESeq2 (v1.42.1) (101) package. For differential expression analysis, the design formula “design = ~batch + Condition” was used to correct for batch effect. For plotting PCA plots and heatmaps, counts were transformed using variance-stabilized transformation (vst) followed by the removal of batch effect using limma (v3.58.1) package (102, 103). DEGs were defined as log2 fold change ≤ −2 or ≥ +2 and adjusted *P* value ≤ 0.01. For analyzing DEGs for naïve vs LPS-stimulated macrophages from publicly available data sets (42, 43), DEGs were defined as log2 fold change ≤ −1 or ≥ +1 and adjusted *P* value ≤ 0.05. DEG analysis of RNA-seq is found in Table S3. Pathway analysis was performed using the DAVID analysis tool (104).

For the analysis of counterregulated genes between WA314ΔYopQ, WA314ΔYopM, and WA314ΔYopP DEGs between WAC and WA314 6 h were used and further selected if the same genes were upregulated by WA314ΔYopP vs WA314 6 h (log2 fold change ≥ +1) but downregulated by WA314ΔYopM or WA314ΔYopQ vs WA314 6 h (log2 fold change ≤ −1) or if the same genes were downregulated by WA314ΔYopP vs WA314 6 h (log2 fold change ≤ −1) but upregulated by WA314ΔYopM or WA314ΔYopQ vs WA314 6 h (log2 fold change ≥ +1).

### TF motif analysis

TF motif enrichment for known motifs was performed using the HOMER package (105). Command *findMotifs.pl* was used, and a list of gene symbols was supplied as input. Motifs were searched in the region 400 bp upstream and 100 bp downstream of the TSS by specifying parameters *-start −400 -end 100*. For the presentation of enriched TF motifs, results from known motifs were used.

### Boxplots

Boxplots were generated using ggplot2 (3.5.1) in RStudio (2024.09.1+394). Boxes encompass the 25th to 75th percentile changes. Whiskers extend to the 10th and 19th percentiles. Outliers are depicted with black dots. The central horizontal bar indicates the median.

### Chromatin immunoprecipitation and sequencing

Chromatin immunoprecipitation and sequencing (ChIP-seq) with formaldehyde crosslinking was performed as described previously (28). Macrophages (3–10 × 10^6^ cells per condition, 2–3 biological replicates/macrophage donors per condition) were washed once with warm PBS and incubated for 30 min at 37°C with Accutase (eBioscience) to detach the cells. For ChIP, BSA-blocked ChIP-grade protein A/G magnetic beads (Thermo Fisher Scientific) were added to the chromatin and antibody mixture and incubated for 2 h at 4°C, rotating to bind chromatin-antibody complexes. Samples were incubated for ~3 min with a magnetic stand to ensure attachment of beads to the magnet and mixed by pipetting during the wash steps. Eluted DNA was subjected to ChIP-seq library preparation. Input chromatin DNA was prepared from 1/4 of the chromatin amount used for ChIP. Antibodies used for ChIP were anti-H3S10ph (Invitrogen, 9H12L10, 6 µL [3 µg] per ChIP) and anti-H3K9me3S10ph (abcam, ab5819, 2 µg per ChIP).

ChIP-seq libraries were constructed with 1–10 ng of ChIP DNA or input control as a starting material. Libraries were generated using the NEXTflex ChIP-Seq Kit (Bioo Scientific) as per the manufacturer’s recommendations. Concentrations of all samples were measured with a Qubit Fluorometer (Thermo Fisher Scientific), and the fragment length distribution of the final libraries was analyzed with the DNA High Sensitivity Chip on an Agilent 2100 Bioanalyzer (Agilent Technologies). All samples were normalized to 2 nM and pooled equimolar. The library pool was sequenced on the NextSeq500 (Illumina) with 1 × 75 bp and a total of approximately 20–40 million reads per sample.

### ChIP-seq analysis

Raw FASTQ files from ChIP sequencing were processed using the nf-core/chipseq pipeline (v1.0.0) (https://nf-co.re/chipseq/) within the nf-core framework (96). The pipeline was executed with Nextflow v0.26.4 (97), starting with TrimGalore (http://www.bioinformatics.babraham.ac.uk/projects/trim_galore/) to trim low-quality bases (Phred score cutoff of 20) and adapter sequences. The trimmed reads were then aligned to the GRCh37 human genome using BWA (v0.7.12) (106), generating an alignment format. Samtools (107) was used to perform manipulations on the alignment format, such as sorting, indexing, and format conversions. To remove duplicate reads, Picard (https://broadinstitute.github.io/picard/) was applied, and BEDTools (108) was used to generate BED files from the aligned data.

For defining H3K9me3 regions in macrophages from publicly available data (42), SICER2 (109) was used with input ChIP-seq data as a control. Peaks were filtered to exclude blacklisted regions (110) and regions with *P* values larger than 0.05 and regions with fold changes less than 2. The ChIP-seq signal was plotted using EaSeq (111) (https://easeq.net) and IGV (112).

For analysis of chromatin states using ChromHMM analysis (57, 58), bed files were binarized using a bin size of 1,000 bp. A different number of emissions (chromatin states) was tested, and seven emissions were selected to depict all unique chromatin states.

ChIP-seq data have been deposited in the ArrayExpress database at EMBL-EBI (www.ebi.ac.uk/arrayexpress) under accession number E-MTAB-14995.

## SUPPLEMENTAL MATERIAL

Supplemental Figures mbio.02547-25-s0001.pdf Figures S1 to S5.

Tables S1 to S3 mbio.02547-25-s0002.xlsx Strains, RNA-seq samples, and DEG analysis with DESeq2.

Table S4 mbio.02547-25-s0003.csv vst normalized counts from RNA-seq.

Tables S5 to S7 mbio.02547-25-s0004.xlsx vst counts and clustering from RNA-seq.

Movie S1 mbio.02547-25-s0005.mp4 Representative videos (Fem1, 3 fpm) of human macrophages not infected (mock) or infected with indicated strains at an MOI of 50 for 2 h and loaded with Cal520-AM and FuraRed-AM to visualize intracellular Ca^2+^ fluctuations. Stimulation with 1µM thapsigargin at 120 mpi was used as a positive control. Scale bar, 100 µm. Calibration bar, fluorescence intensity (Fem1) (a.u.). Time stamp, mm:ss.

ASM does not own the copyrights to Supplemental Material that may be linked to, or accessed through, an article. The authors have granted ASM a non-exclusive, world-wide license to publish the Supplemental Material files. Please contact the corresponding author directly for reuse.
