# Supplementary material for: Immune activation of primary human macrophages is suppressed by the coordinated action of Yersinia effectors
Source: mBio. 2025 Nov 24;17(1):e02547-25. doi: 10.1128/mbio.02547-25 (PMC12802187; doi:10.1128/mbio.02547-25)
Supplement: Supplemental Figures — Figures S1 to S5. [file mbio.02547-25-s0001.pdf]

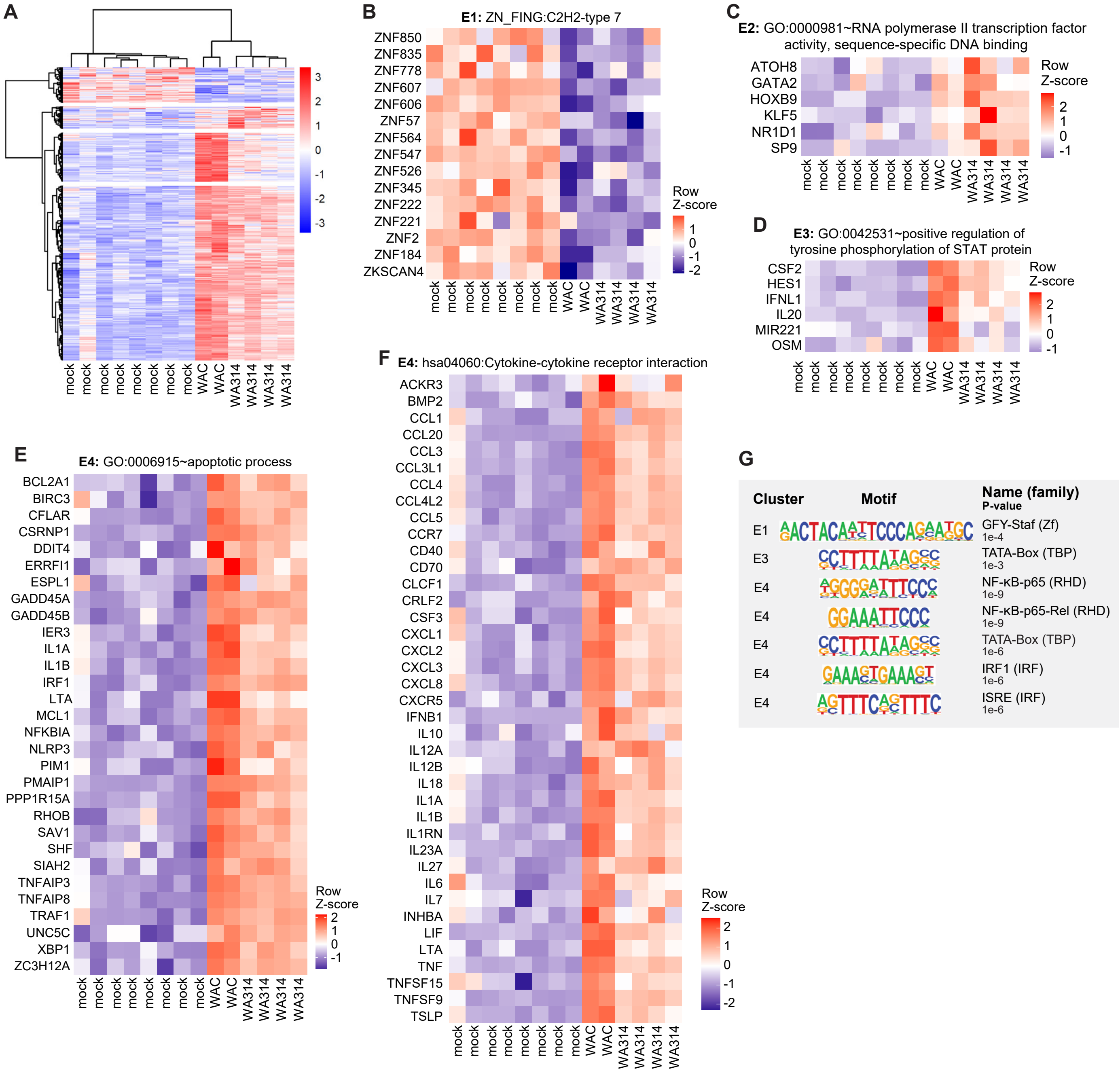

Figure S1:
A: Clustered heatmap of all DEGs for comparisons between mock, WAC and WA314 infected macrophages after 1.5 h of infection showing all replicates used in the analysis (see also Table S2). Clustering showed 4 major clusters. Gene vst counts were row-scaled (row Z-score).
B: Heatmap of row scaled RNA-seq vst counts for genes belonging to Zinc finger genes from the C2H2 type 7 enriched in cluster E1.
C: Heatmap of row scaled RNA-seq vst counts for genes belonging to the RNA polymerase II transcription factor activity, sequence-specific DNA binding pathway enriched in cluster E2.
D: Heatmap of row scaled RNA-seq vst counts for genes belonging to the positive regulation of tyrosine phosphorylation of STAT protein pathway enriched in cluster E3.
E: Heatmap of row scaled RNA-seq vst counts for genes belonging to the apoptotic process pathway enriched in cluster E4.
F: Heatmap of row scaled RNA-seq vst counts for genes belonging to the cytokine-cytokine receptor interaction pathway enriched in cluster E4.
G: Representative enriched transcription factor motifs in genes from clusters E1 - E4 (Fig 2A-C).
Vst counts for generation of heatmaps can be found in the Table S4.

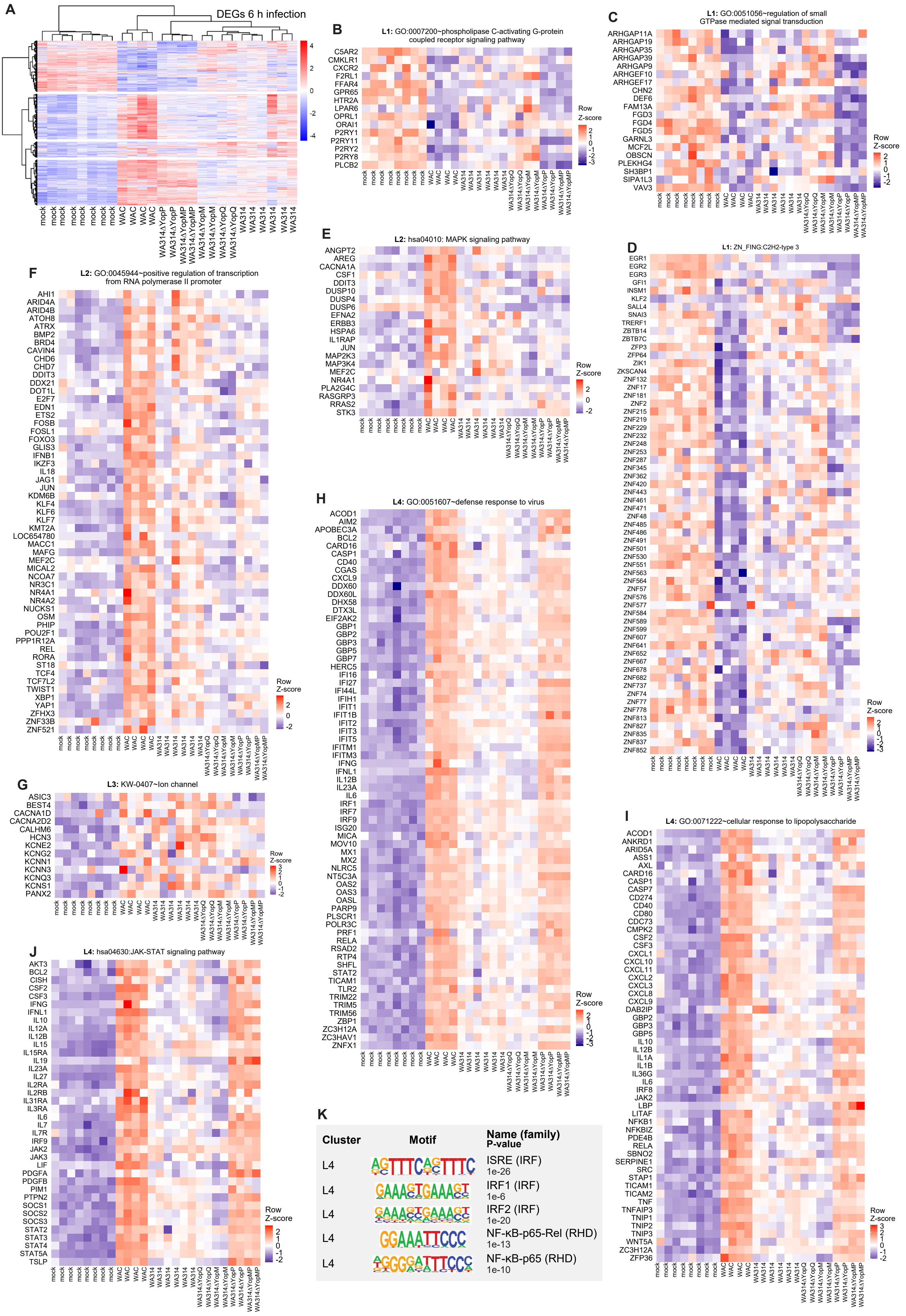

A
DEGs 6 h infection
4
2
0
-2
-4
mock mock mock mock mock mock mock mock WAC WAC WAC WAC WA314ΔYopP WA314ΔYopP WA314ΔYopMP WA314ΔYopMP WA314ΔYopM WA314ΔYopM WA314ΔYopQ WA314ΔYopQ WA314 WA314 WA314 WA314 WA314 WA314
B
L1: GO:0007200~phospholipase C-activating G-protein coupled receptor signaling pathway
C5AR2 CMKLR1 CXCR2 F2RL1 FFAR4 GPR65 HTR2A LPAR6 OPRL1 ORAI1 P2RY1 P2RY11 P2RY2 P2RY8 PLCB2
Row Z-score
C
L1: GO:0051056~regulation of small GTPase mediated signal transduction
ARHGAP11A ARHGAP19 ARHGAP35 ARHGAP39 ARHGAP9 ARHGEF10 ARHGEF17 CHN2 DEF6 FAM13A FGD3 FGD4 FGD5 GARNL3 MCF2L OBSCN PLEKHG4 SH3BP1 SIPA1L3 VAV3
Row Z-score
D
L1: ZN_FING:C2H2-type 3
EGR1 EGR2 EGR3 GFI1 INSM1 KLF2 SALL4 SNAI3 TRERF1 ZBTB14 ZBTB7C ZFP3 ZFP64 ZIK1 ZKSCAN4 ZNF132 ZNF17 ZNF181 ZNF2 ZNF215 ZNF219 ZNF229 ZNF232 ZNF248 ZNF253 ZNF287 ZNF345 ZNF362 ZNF420 ZNF443 ZNF461 ZNF471 ZNF48 ZNF485 ZNF486 ZNF491 ZNF501 ZNF530 ZNF551 ZNF563 ZNF564 ZNF57 ZNF576 ZNF577 ZNF584 ZNF589 ZNF599 ZNF607 ZNF641 ZNF652 ZNF667 ZNF678 ZNF682 ZNF737 ZNF74 ZNF77 ZNF778 ZNF813 ZNF827 ZNF835 ZNF837 ZNF852
Row Z-score
E
L2: hsa04010: MAPK signaling pathway
ANGPT2 AREG CACNA1A CSF1 DDIT3 DUSP10 DUSP4 DUSP6 EFNA2 ERBB3 HSPA6 IL1RAP JUN MAP2K3 MAP3K4 MEF2C NR4A1 PLA2G4C RASGRP3 RRAS2 STK3
Row Z-score
F
L2: GO:0045944~positive regulation of transcription from RNA polymerase II promoter
AHI1 ARID4A ARID4B ATOH8 ATRX BMP2 BRD4 CAVIN4 CHD6 CHD7 DDIT3 DDX21 DOT1L E2F7 EDN1 ETS2 FOSB FOSL1 FOXO3 GLIS3 IFNB1 IKZF3 IL18 JAG1 JUN KDM6B KLF4 KLF6 KLF7 KMT2A LOC654780 MACC1 MAFG MEF2C MICAL2 NCOA7 NR3C1 NR4A1 NR4A2 NUCKS1 OSM PHIP POU2F1 PPP1R12A REL RORA ST18 TCF4 TCF7L2 TWIST1 XBP1 YAP1 ZFHX3 ZNF33B ZNF521
Row Z-score
G
L3: KW-0407~Ion channel
ASIC3 BEST4 CACNA1D CACNA2D2 CALHM6 HCN3 KCNE2 KCNG2 KCNN1 KCNN3 KCNQ3 KCNS1 PANX2
Row Z-score
H
L4: GO:0051607~defense response to virus
ACOD1 AIM2 APOBEC3A BCL2 CARD16 CASP1 CD40 CGAS CXCL9 DDX60 DDX60L DHX58 DTX3L EIF2AK2 GBP1 GBP2 GBP3 GBP5 GBP7 HERC5 IFI16 IFI27 IFI44L IFIH1 IFIT1 IFIT1B IFIT2 IFIT3 IFIT5 IFITM1 IFITM3 IFNG IFNL1 IL12B IL23A IL6 IRF1 IRF7 IRF9 ISG20 MICA MOV10 MX1 MX2 NLRC5 NT5C3A OAS2 OAS3 OASL PARP9 PLSCR1 POLR3C PRF1 RELA RSAD2 RTP4 SHFL STAT2 TICAM1 TLR2 TRIM22 TRIM5 TRIM56 ZBP1 ZC3H12A ZC3HAV1 ZNFX1
Row Z-score
I
L4: GO:0071222~cellular response to lipopolysaccharide
ACOD1 ANKRD1 ARID5A ASS1 AXL CARD16 CASP1 CASP7 CD274 CD40 CD80 CDC73 CMPK2 CSF2 CSF3 CXCL1 CXCL10 CXCL11 CXCL2 CXCL3 CXCL8 CXCL9 DAB2IP GBP2 GBP3 GBP5 IL10 IL12B IL1A IL1B IL36G IL6 IRF8 JAK2 LBP LITAF NFKB1 NFKBIZ PDE4B RELA SBNO2 SERPINE1 SRC STAP1 TICAM1 TICAM2 TNF TNFAIP3 TNIP1 TNIP2 TNIP3 WNT5A ZC3H12A ZFP36
Row Z-score
J
L4: hsa04630:JAK-STAT signaling pathway
AKT3 BCL2 CISH CSF2 CSF3 IFNG IFNL1 IL10 IL12A IL12B IL15 IL15RA IL19 IL23A IL27 IL2RA IL2RB IL31RA IL3RA IL6 IL7 IL7R IRF9 JAK2 JAK3 LIF PDGFA PDGFB PIM1 PTPN2 SOCS1 SOCS2 SOCS3 STAT2 STAT3 STAT4 STAT5A TSLP
Row Z-score
mock WAC WA314 WA314ΔYopQ WA314ΔYopM WA314ΔYopP WA314ΔYopMP
K
Cluster | Motif | Name (family) P-value
L4 | AGTTTCAGTTTC | ISRE (IRF) 1e-26
L4 | GAAAGTGAAAGT | IRF1 (IRF) 1e-6
L4 | GAAAGTGAAAGT | IRF2 (IRF) 1e-20
L4 | GGAAATTCCC | NF-κB-p65-Rel (RHD) 1e-13
L4 | AGGGGATTTCCC | NF-κB-p65 (RHD) 1e-10

Figure S2:
A: Clustered Heatmap of all DEGs for comparisons between mock, WAC, WA314, WA314ΔYopP, WA314ΔYopMP, WA314ΔYopM, WA314ΔYopQ infected macrophages after 6 h of infection showing all replicates used in the analysis. Clustering showed 4 major clusters. Gene vst counts were row-scaled (row Z-score).
B: Heatmap of row scaled RNA-seq vst counts for genes belonging to the phospholipase C-activating G-protein coupled receptor signaling pathway enriched in cluster L1 from all replicates used in this study.
C: Heatmap of row scaled RNA-seq vst counts for genes belonging to the regulation of small GTPase mediated signal transduction pathway enriched in cluster L1 from all replicates used in this study.
D: Heatmap of row scaled RNA-seq vst counts for genes belonging to Zinc finger genes from the C2H2 type 3 enriched in cluster L1 from all replicates used in this study.
E: Heatmap of row scaled RNA-seq vst counts for genes belonging to MAPK signaling pathway enriched in cluster L2 from all replicates used in this study.
F: Heatmap of row scaled RNA-seq vst counts for genes belonging to the positive regulation of transcription from RNA polymerase II promotor pathway enriched in cluster L2 from all replicates used in this study.
G: Heatmap of row scaled RNA-seq vst counts for genes belonging to the group of ion channels enriched in cluster L3 from all replicates used in this study.
H: Heatmap of row scaled RNA-seq vst counts for genes belonging to defence response to virus pathway enriched in cluster L4 from all replicates used in this study.
I: Heatmap of row scaled RNA-seq vst counts for genes belonging to the cellular response to lipopolysaccharide pathway enriched in cluster L4 from all replicates used in this study.
J: Heatmap of row scaled RNA-seq vst counts for genes belonging to the JAK-STAT signaling pathway enriched in cluster L4 from all replicates used in this study.
K: Representative enriched transcription factor motifs in genes from L1 - L4 clusters in Fig 3E.
Vst counts for generation of heatmaps can be found in the Table S4.

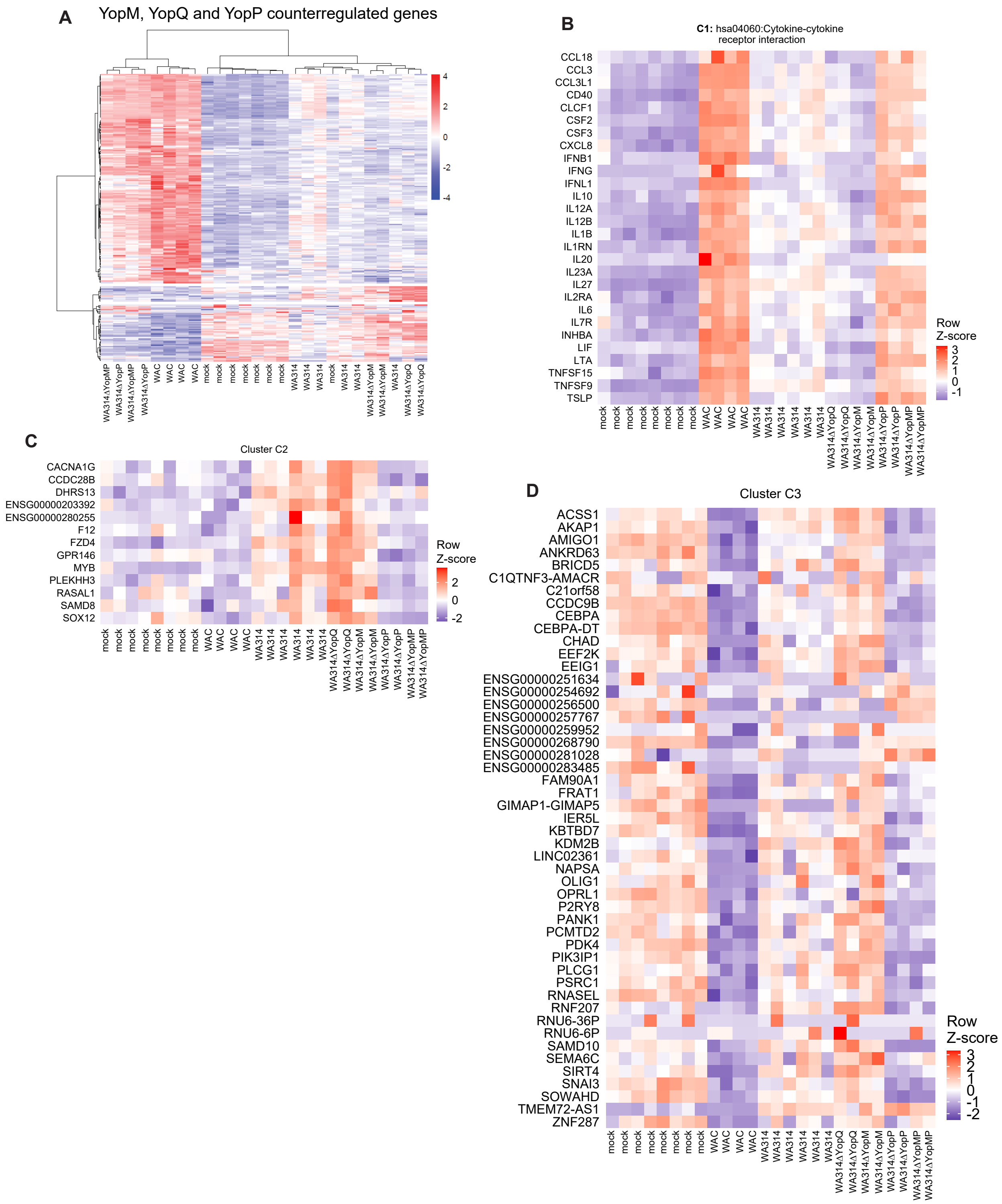

Figure S3:

A: Clustered heatmap of genes counterregulated by YopM, YopP and YopQ in comparisons between WAC- and WA314-infected macrophages (6 h) showing all replicates used for the analysis. Gene vst counts were row-scaled (row Z-score) and clustering identified 3 major clusters.

B: Heatmap of row scaled RNA-seq vst counts for genes belonging to cytokine-cytokine receptor interaction pathway enriched in cluster C1 from all replicates used in this study.

C: Heatmap of row scaled vst counts for genes in cluster C2 showing all replicates used in the study.

D: Heatmap of row scaled vst counts for genes in cluster C3 showing all replicates used in the study.

Vst counts for generation of heatmaps can be found in the Table S4.

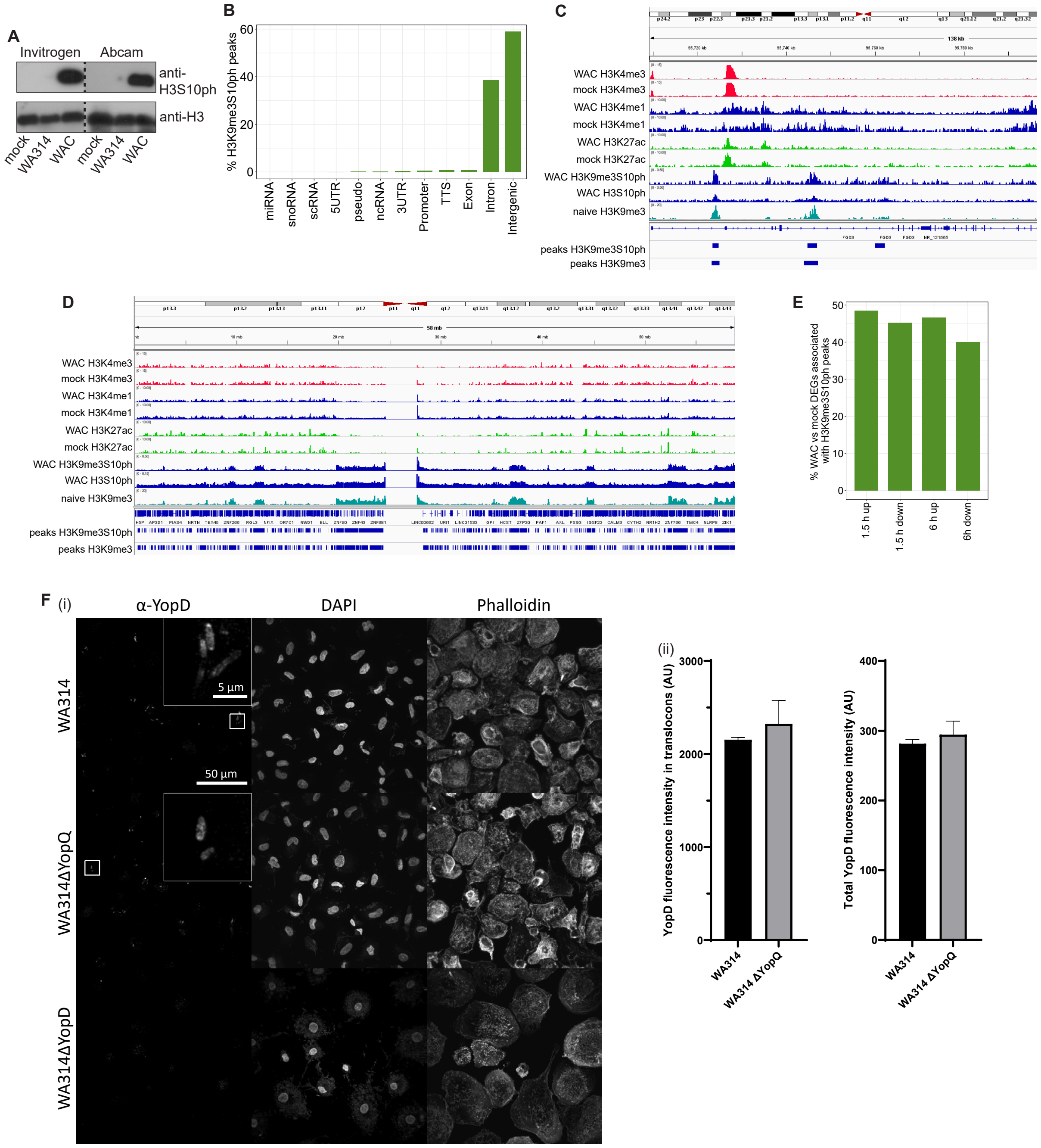

Figure S4:
A: Western blot showing H3S10ph levels with two different anti-H3S10ph antibodies and anti-H3 in macrophages not infected (mock) or infected with WA314 or WAC for 3 h with MOI of 100. H3 bands serve as loading control.
B: Annotation of H3K9meS10ph ChIP-seq peaks to genomic regions from WAC-infected macrophages using homer tool (Heinz et al., 2010).
C and D: Peak tracks of ChIP-seq tag densities for indicated histone modifications in either WAC- or mock-infected macrophages or H3K9me3 peaks from naïve macrophages from publicly available dataset (Novakovic et al., 2016).
E: Percentage of up- or downregulated DEGs for WAC vs mock after 1.5 h or 6h that associate with H3K9me3S10ph peaks. H3K9me3S10ph peaks were assigned to the closest gene using homer tool (Heinz et al., 2010).
F: Comparison of YopD fluorescence intensity in translocons and total YopD fluorescence intensity in macrophages infected with *Y.enterocolitica* wild-type strain WA314 and YopQ deletion mutant WA314ΔYopQ. Macrophages were infected with a MOI of 100 for 60 mins. The translocon protein YopD was stained using a primary α-YopD antibody and a fluorescently labelled secondary antibody. (i) Representative images of fluorescence microscopy of α-YopD antibody stainings during cell infection. WA314ΔYopD infection served as negative control. White boxed areas indicate areas that were enlarged for the zoom-ins. Scale bars: 50 µm in overview images. 5 µm in zoom-ins. (ii) The graph on the left shows the YopD fluorescence intensity in translocons (AU). The graph on the right shows the total YopD fluorescence intensity in the microscopy image (AU). Intensities for YopD fluorescence in translocons are higher, as the mean fluorescent intensity in these measured areas is higher, in comparison to a comparatively lower mean fluorescence given by potentially translocated YopD or background staining for total YopD fluorescence measurements. Bars represent means from three independent experiments with standard deviation indicated by error bars. Translocon formation was consistently not significantly different between WA314 and WA314ΔYopQ.

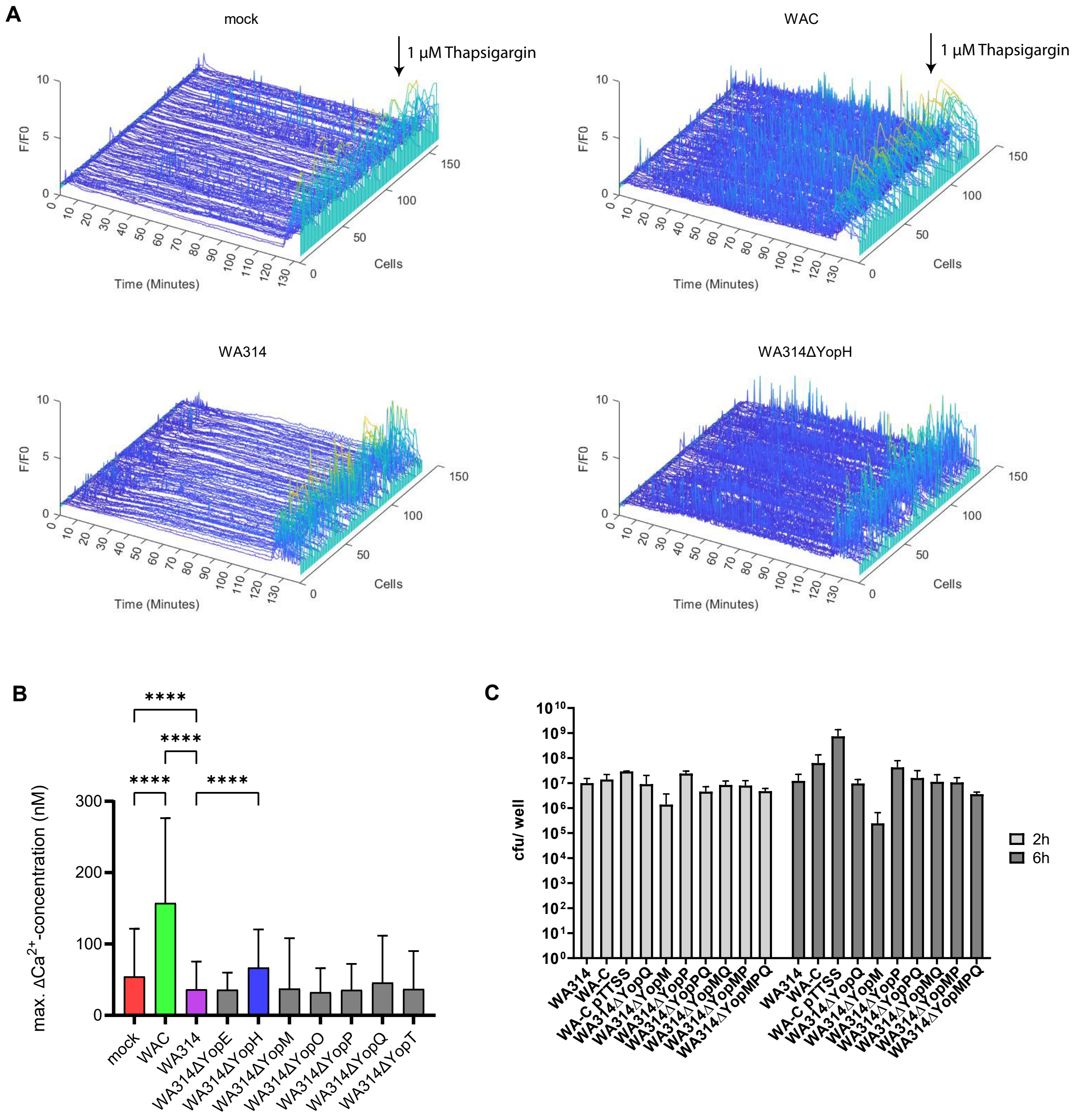

Figure S5:

A: Representative normalized (F_{em1}/F_{em10}) single-cell fluorescence intensity profiles from human macrophages not infected (mock) or infected with indicated strains with a MOI of 50 for 2h and loaded with Cal520-AM and FuraRed-AM. Cells were imaged with 3 frames per min (fpm) for 120 min. Stimulation with 1 µM Thapsigargin at 120 min post infection (mpi) was used as a positive control.

B: Quantification of maximal ΔCa^{2+}-concentration/cell.

C: Measurement of cell associated bacterial load of primary human macrophages infected with *Y. enterocolitica* wildtype and indicated mutant strains. Macrophages were seeded at a density of 2 x 10^5 cells per well and infected with the indicated strains for either 2 h or 6 h with MOI 100. After the indicated time of infection cells were washed, lysed with 0.5 % Digitonin in PBS and serial dilutions of lysate were plated on LB agar plates. Appropriate dilutions were selected and colonies counted after growth at 27 °C for 48 h. The colony forming units (cfu) / well of two technical replicates were calculated. Bars indicate means of three independent experiments with different macrophage donors, standard deviation is indicated as error bars.
